# *SOD1* mutations associated with amyotrophic lateral sclerosis analysis of variant severity

**DOI:** 10.1038/s41598-021-03891-8

**Published:** 2022-01-07

**Authors:** Mariusz Berdyński, Przemysław Miszta, Krzysztof Safranow, Peter M. Andersen, Mitsuya Morita, Sławomir Filipek, Cezary Żekanowski, Magdalena Kuźma-Kozakiewicz

**Affiliations:** 1 Laboratory of Neurogenetics, Department of Neurodegenerative Disorders, Mossakowski Medical Research Institute, Polish Academy of Sciences, Warsaw, Poland; 2 Department of Clinical Sciences, Neurosciences, Umeå University, Umeå, Sweden; 3 Faculty of Chemistry, Biological and Chemical Research Centre, University of Warsaw, Warsaw, Poland; 4 Department of Biochemistry and Medical Chemistry, Pomeranian Medical University, 72 Powstańców Wlkp. Str., 70-111 Szczecin, Poland; 5 Division of Neurology, Department of Internal Medicine, Jichi Medical University, Shimotsuke, Japan; 6 Department of Neurology, Medical University of Warsaw, Warsaw, Poland; 7 Neurodegenerative Diseases Research Group, Medical University of Warsaw, Warsaw, Poland

**Keywords:** Amyotrophic lateral sclerosis, Genetic testing, Amyotrophic lateral sclerosis

## Abstract

Mutations in superoxide dismutase 1 gene (*SOD1*) are linked to amyotrophic lateral sclerosis (ALS), a neurodegenerative disorder predominantly affecting upper and lower motor neurons. The clinical phenotype of ALS shows inter- and intrafamilial heterogeneity. The aim of the study was to analyze the relations between individual *SOD1* mutations and the clinical presentation using in silico methods to assess the *SOD1* mutations severity. We identified *SOD1* causative variants in a group of 915 prospectively tested consecutive Polish ALS patients from a neuromuscular clinical center, performed molecular modeling of mutated *SOD1* proteins and in silico analysis of mutation impact on clinical phenotype and survival analysis of associations between mutations and hazard of clinical end-points. Fifteen *SOD1* mutations were identified in 21.1% familial and 2.3% sporadic ALS cases. Their effects on SOD1 protein structure and functioning inferred from molecular modeling and in silico analyses correlate well with the clinical data. Molecular modeling results support the hypothesis that folding intermediates rather than mature SOD1 protein give rise to the source of cytotoxic conformations in ALS. Significant associations between type of mutation and clinical end-points were found.

## Introduction

Amyotrophic lateral sclerosis (OMIM:105400) is a heterogeneous severe neurodegenerative disorder the hallmark of which is an adult-onset loss of upper and lower motor neurons. It leads to a progressive paresis and atrophy of skeletal muscles resulting in quadriplegia and fatal respiratory failure. Approximately 90–95% of patients do not have affected first-degree relatives and are described as sporadic cases (sporadic ALS, sALS)^1^, and ca. 10% show a familial predisposition (fALS) with Mendelian or non-Mendelian patterns of inheritance^2^. Since 1993, mutations in more than forty genes have been reported to associate with ALS, the most frequent are those in the *SOD1* gene encoding the essential antioxidant enzyme Cu, Zn superoxide dismutase (http://alsod.iop.kcl.ac.uk/)^3,4^. Coding sequences (cds) *SOD1* mutations have been found in ALS patients from all over the world. However, the distribution of *SOD1* mutations differs markedly even among apparently similar populations (i.e., Netherlands and Belgium, Ireland and England) and the same mutations in different populations can be associated with distinct clinical presentations. The clinical phenotype is highly variable and patients with a particular *SOD1* mutation show intrafamilial differences in the severity of symptoms and the speed of disease progression^5^. Notably, it is believed that the pathogenicity of *SOD1* mutations is not due to a lack of functional protein but rather to the accumulation of its misfolded aggregates^6^.

It is still unclear whether all ALS-related *SOD1* mutations are in fact causative, co-causative, modifying or simply accompanying variants. A prospective gene therapy targeting SOD1 expression or a pharmacotherapy aimed at elimination of misfolded SOD1 protein can only be based on a detailed understanding of the molecular mechanisms of pathogenesis of individual *SOD1* mutations^7^.

To address the above issues, we determined *SOD1* mutations in a large group of ALS patients (n = 915) and predicted their impact on SOD1 structure and functioning using molecular modeling and prioritization algorithms. These predictions were compared with the severity of ALS presentation in individual patients. A significant correspondence was found between molecular and clinical data.

## Methods

### Subjects

A total of 915 patients with ALS (n = 855 unrelated sALS and 57 probands from fALS families, 6.2% FALS) were diagnosed at the Department of Neurology, the Medical University of Warsaw between 2006 and 2018 and followed till 2021. The patient clinical data was prospectively analyzed and the blood was withdrawn at the time of the first clinical assessment. The patients were further regularly followed at the same out-patient clinic, and examined up to five times during the disease course. All patients were Caucasians of a Polish origin and met the El Escorial criteria for clinically possible, probable or definite ALS^8^.

### Mutation screening and variant analysis

DNA was isolated from peripheral blood leukocytes using standard methods, and all exons with flanking intronic regions of the *SOD1* gene were sequenced as described previously^9^.

The Ensembl Variant Effect Predictor (https://www.ensembl.org/Homo_sapiens/Tools/VEP) was used to annotate genomic variants^10^. Human Splicing Finder 3.0 (HSF3), EX-SKIP, and BDGP Splice Site Prediction by Neural Network webtools were used to predict the influence of detected variants on pre-mRNA splicing^11–13^. ConSurf (https://consurf.tau.ac.il/) was used to analyze amino acid sequence conservation^14^. PredictSNP (https://loschmidt.chemi.muni.cz/predictsnp/) and NetDiseaseSNP (http://www.cbs.dtu.dk/services/NetDiseaseSNP/) were used to predict the impact of mutations on the function of SOD1 protein^15,16^. Aggrescan (http://bioinf.uab.es/aggrescan/), TANGO (http://tango.crg.es/) and Aggrescan3D 2.0 (http://biocomp.chem.uw.edu.pl/A3D2/) software were used to predict the tendency for aggregation of mutated proteins^17–19^.

### Molecular modeling

The crystal structure of human SOD1 was taken from the Protein Data Bank (PDB id:2C9V)^20^. SOD1 is a compact homodimer with each subunit of 153 amino acids forming a β-barrel structure stabilized by a C57–C146 disulfide bridge and a zinc ion in the active site. The two subunits are held together by strong hydrophobic forces making SOD1 one of the most compact and stable proteins. Each subunit also contains a copper ion undergoing alternative oxidation–reduction in the course of the dismutation of O_2_^·−^ to O_2_ and H_2_O_2_. The metal ions are bound by the side chains of H46, H48, H63, H71, H80, D83, and H120. The force field parameters and partial charges for the metal ion binding sites were calculated following earlier quantum chemical calculations on similar systems^21,22^. To impose new parameters, a patch script for the topology file was constructed to model proper interactions between the metal ions and adjacent amino acid atoms. Energy minimization and molecular dynamic (MD) simulations were performed in the NAMD program version 2.10 using all-atom force field CHARMM27^23^. The protein dimer was simulated in the TIP3P water box with dimensions of 6.2 nm × 6.2 nm × 9.5 nm, which contained 37,000 atoms in total. Four sodium ions were also included in solution to maintain neutrality of the system. The native protein as well as its 12 mutant variants were initially subjected to 10,000 steps of energy minimization and then a 10-ns MD equilibration with temperature increasing from 20 to 298 K. For each investigated system a 20-ns MD simulation was performed. All MD simulations were conducted using Langevin (stochastic) dynamics^24^ used as default in the NAMD program. The friction coefficient of 5 ps^−1^ was used and the temperature was set to 298 K. Nonbonded interactions were dampened by employing a switching function for van der Waals and electrostatic interactions using a cutoff of 1.6 nm. All bond lengths were constrained using the SHAKE algorithm^25^, therefore a longer time step of 2 fs was applied. The modeled molecular structures were visualized YASARA Structure v.16.1.2.

### Statistical analyses

Associations between *SOD1* mutations carried by ALS patients and theirs clinical phenotype were studied with survival analysis methods. Four clinically relevant end-points were defined to estimate progression of the disease: wheelchair-bound (loss of walking capacity), bulbar involvement (speech or swallowing impairment), respiratory insufficiency and death (overall survival). All available information from patients’ records were used to find whether the chosen end-point happened (if yes the observation was considered “complete”, if no it was “censored”) and what was the time from the onset of ALS symptoms (defined as the first muscle paresis) to the achievement of the selected end-point (for complete observations) or to the end of observation (for censored observations). Kaplan–Meier curves showing survival till each end-point for patients stratified according to specific *SOD1* mutations or a bioinformatics parameter common for a group of mutations were compared with log-rank test for two curves or with chi-square test for more than two curves. Cox proportional hazards regression model was used to estimate associations between survival and quantitative Consurf parameter and calculate hazard ratio (HR) and its 95% confidence interval. Only mutations found in at least four subjects with available clinical data were subjected to direct comparisons. Age of ALS onset was compared between mutations with Mann–Whitney test. Site of ALS onset and clinical phenotype were compared between mutations with Fisher exact test. P < 0.05 was considered statistically significant. Statistica 13 program was used for statistical calculations.

### Ethics approval and consent to participate

A written informed consent has been obtained from all study participants. The study has been approved by the Bioethics Committee of the Medical University of Warsaw, Poland (KB 157/2006, KB 52/2012, KB/163/2015), in compliance with the Declaration of Helsinki (BMJ 1991; 302:1194), national legislation and the Code of Ethical Principles for Medical Research Involving Human Subjects of the World Medical Association.

## Results

### Identifications of variants in SOD1

Sequencing revealed a total of 23 *SOD1* variants, fifteen of which were in the protein coding sequence (for details see Supplementary Table S1). Among the 15 variants, all but one (p.W32*) have previously been described as causative of ALS. Three variants (c.-64C > T, c.-6G > T and c.*248A > C) lied in untranslated gene regions. Three other variants in the flanking intronic sequences: c.239 + 34A > C (MAF = 0.039 according to ExAC database), c.72 + 19G > A (MAF < 0.001), and a deletion of 7 bp in intron 2 previously described in families with keratoconus (MAF = 0.003)^26,27^. All the variants except one were in a heterozygous state: two ALS patients were homozygous and two heterozygous for the c.98G > A (p.D90A) *SOD1* mutation. Variants c.10A > G (p.K3E), c.124G > A (p.G41S), c.260A > G (p.N86S), c.272A > C (p.D90A), c.418A > G (p.N139D) and c.434T > C (p.L144S) were identified in several patients with either sALS and/or fALS. All other variants but one (common c.239 + 34A > C) were identified in single patients. The most frequent mutations were K3E (5.3% fALS—3 families/57 familial index cases, 0.5% sALS—4 cases/854 sALS cases), L144S (8.8% fALS-5 families/57 familial index cases, 0.2%—2 cases/854 sALS cases), and D90A (0% fALS, 0.5%—4 cases/854 sALS cases respectively) followed by G41S, L126* and N139D. The overall frequency of *SOD1* mutations in the study group was 3.5%—32/915 cases, 21.1% fALS—12 families/57 familial index cases, 2.3% sALS—30 cases/854 sALS cases).

### Clinical characteristics of the patients

Fifteen different *SOD1* coding-sequence (cds) mutations were identified in 12 fALS and 20 sALS cases. Based on the medical documentation and familial anamnesis we gathered clinical data on additional 32 FALS subjects from the affected families. In total we analyzed clinical data of 64 patients with *SOD1* cds mutations. Their general demographics are summarized in Table 1.

**Table 1:** Demographic data of patients with amyotrophic lateral sclerosis.

ALS patients	Male:female ratio	Age at onset (years) Median; mean ± SD (range)	Diagnosis delay (months) Median; mean ± SD (range)
All n = 918	1.07	56 55.2 ± 12.7 (18–86)	11 13.7 ± 10.1 (1–48)
Without *SOD1* mutations n = 854	1.1	57 55.6 ± 12.7 (18–86)	11 13.6 ± 10.1 (1–48)
*SOD1* mutation carriers n = 64 (44 fALS, 20 sALS)	0.7	51 50.2 ± 10.6 (21–75)	10 16.6 ± 14.9 (2–72)

Among patietns with *SOD1* mutations, the classic ALS phenotype was observed in 56% of patients (both upper and lower motor neuron involvement; UMN, LMN), 41% showed progressive muscle atrophy (PMA, isolated signs of LMN), and 3% (n = 1) a mixed ALS-MSA-P (multiple system atrophy-parkinsonism) phenotype. The signs of the UMN damage included pseudobulbar syndrome, spasticity, and exaggerated reflexes and pathological signs. The LMN damage presented as muscle wasting, fasciculations, flaccid muscle tone and diminished/absent reflexes. In 88.1% of the patients the first symptoms occurred in the lower limbs, in 9.5% in the upper limbs, and in 2.4% in the bulbar-innervated muscles. The median survival was at least 84 months (mean 105.0 ± 69.4; range 12–312), while the tracheostomy-free survival was at least 36 months (mean 73.8 ± 75.8; range 11–312 months), since 17 patients were still alive at the time of analysis. Detailed clinical characteristics of the patients with an *SOD1* coding-sequence mutation are presented in Table 2.

**Table 2:** Clinical characteristics of ALS patients (n = 52) with *SOD1* cds mutations.

Mutation	No. of fALS families (cases)/sALS cases with identified mutations	Age at onset ± SD, range, (years)	M:F	Diagnosis delay (months)	Site of onset	Clinical phenotype	Bulbar involvement during disease course (months since onset ± SD, range, %)	Wheelchair-bound (months since onset ± SD, range, %)	IV-free survival (months since onset ± SD, range)
K3E	3 fALS (n = 14), 4 sALS	53.3 ± 8.1, 36–68 (n = 16)	61% M, 39% F	9.2 ± 3.2, 6–26 (n = 8)	94% LL, 6% UL (n = 16)	80% classic ALS, 20% PMA (n = 10)	32.8 ± 25.8, 5–96 (n = 9), 78.6% yes	33.4 ± 18.9, 12–96 (n = 12), 100% yes	90.3 ± 68.9, 18–180 (n = 16)
A4V	1 sALS	56	F	10	LL	Classic ALS	12	13	18
W32*	1 sALS	41	M	8	LL	Classic ALS	35	24	56
G37R	1 sALS	40	F	4	UL	Classic ALS	8	14	> 48 months
G41S	1 fALS (n = 5)	50.2 ± 11.6, 40–69 (n = 5)	40% M 60% F	7.0 ± 1.0, 6–8 (n = 3)	60% LL, 40% UL (n = 5)	75% classic ALS, 25% PMA (n = 4)	13.0 ± 8.5, 7–19 (n = 2)	NA	15.0 ± 3.6, 11–20 (n = 5)
G72S	1 sALS	52	M	20	LL	PMA	NA	38	> 38
N86S	2 sALS	45 ± 33.9, 21–70 (n = 2)	1:1	15.5 ± 7.8, 10–21 (n = 2)	50% UL, 50% LL	100% PMA	50%	NA	Mean 65.5 ± 41.1, 35–96, (n = 2)
D90A	2 sALS heterozygote	57.1 ± 12.7, 40–75 (n = 2)	100% F	8 (n = 1)	50% LL, 50% bulbar, (n = 2)	100% classic ALS	48 ± 67.9, 0–96 (n = 2)	NA	60.5 ± 50.2, 25–96 (n = 2)
	2 sALS homozygote	47 (n = 1)	100% F	19 (n = 1)	100% LL (n = 1)	100% classic ALS (LMN > UMN)	48 (n = 1)	NA	> 132 (n = 1)
G93C	1 sALS	32	F	72	LL	PMA	96	96	> 96
S105L	1 sALS	42	M	12	LL	PMA	10	Yes	36
D109Y	1 sALS	59	M	33	LL	ALS-MSA-P	No	No	> 52
C111Y	1 sALS	43	M	8	UL	Classic ALS	NA	NA	NA
L126*	1 fALS (n = 4)	49.7 ± 14.3, 34–62 (n = 3)	75% F, 25% M	19.0 ± 14.8, 9–36 (n = 3)	100% LL	75% PMA, 25% classic ALS	50%	26 (n = 1)	132.0 ± 135.8, 36–228 (n = 2)
N139D	2 fALS (n = 4)	57.0 ± 7.0, 49–67 (n = 2)	100% F	9 (n = 1)	100% LL (n = 3)	100% PMA (n = 3)	33% (n = 3)	12 (n = 1)	99 ± 43.6, 24–144 (n = 3)
L144S	5 fALS families (n = 17), 2 sALS	45.8 ± 9.8, 29–63 (n = 14)	84% F, 16%M	23.9 ± 14.7, 2–45, (n = 7)	100% LL, n = 9	50% classic ALS, 50% PMA (n = 8)	67.5 ± 6.3, 63–72 (n = 2/14, 14%)	87.2.2 ± 50.3, 36–156 (n = 6)	125.0. ± 68.4, 36–219 (n = 14)

### Molecular modeling

The structures of the SOD1 wild-type dimer and 14 mutant proteins were subjected to 20-ns all-atom MD simulations in a water environment, which is enough to equilibrate the system and form new interactions after a mutation is introduced. A comparison of the obtained mutant structures with the WT one, revealed how the mutation affected the structure of the SOD1 dimer and also how it could alter potential interactions with other proteins (Table 3, Fig. 1, Supplementary Figs. S1–S15).

**Table 3:**
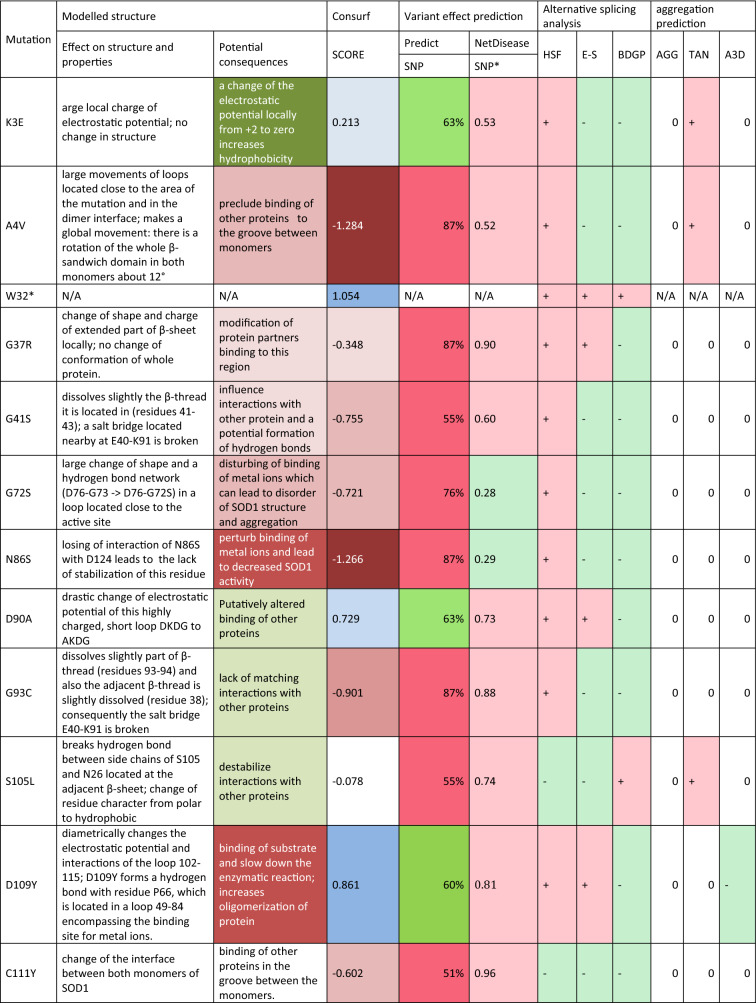

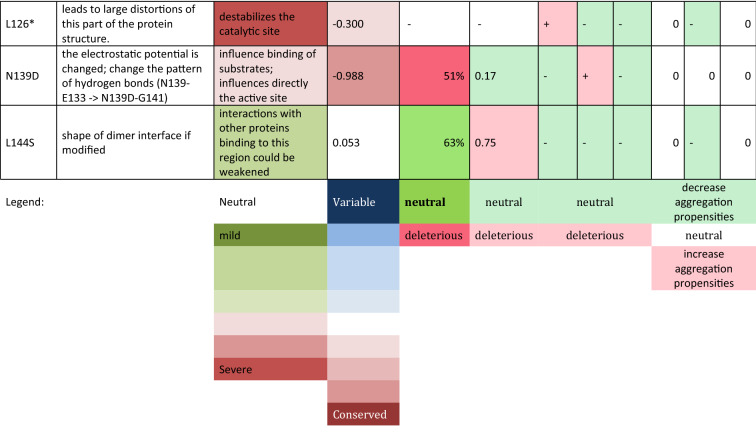
Impact of mutations on SOD1 protein.

Alternative splicing analysis—probability of altering splicing by mutated allel vs. reference (+ higher; −no changes) (details Supplementary Table S4) *HSF* Human Splice Finder interperatation, *E-S* EX-SKIP, *BDGP* BDGP splice site, *Aggregation prediction* the tendency for aggregation of mutated allel vs reference (+ higher, − lower) (details Supplementary Table S5, Supplementary File 2, Supplementary File 3) *AGG* AGGRESCAN, *TAN* TANGO, *A3D* Aggrescan3D.

**Figure 1:**
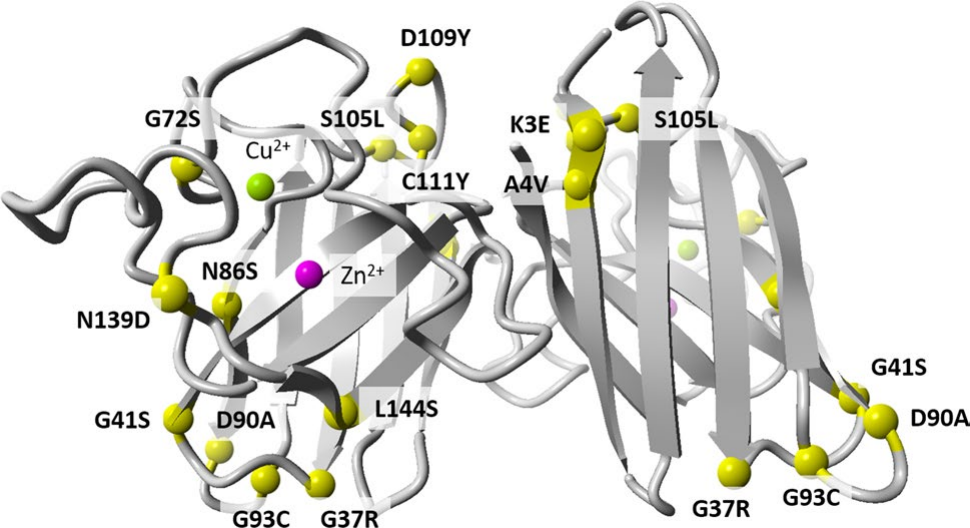
Comparison of protein structure of reference and mutant SOD1. The following mutants were modelled basing on the reference structure (PDB id:2C9V): K3E, A4V, G41S, N86S, D90A, G93C, S105L, D109Y, C111Y, L126*, N139D, and L144S.

### Analysis of coding sequence conservation

Most of the identified mutations affected evolutionarily conserved residues of SOD1. Positions 32 and 109 are evolutionarily variable, and two other variable positions, D90 and L144, were the most frequently mutated in the analyzed group in both familial and sporadic cases. For details see Supplementary Table S2.

### Predicting the functional impact of mutation

To infer probable functional consequences of the *SOD1* mutations we analyzed them with PredictSNP software, which is a consensus classifier of eight commonly used tools: MAPP, nsSNPAnalyzer, PANTHER, PhD-SNP, PolyPhen-1, PolyPhen-2, SIFT, and SNAP. The most common mutations identified in the analyzed group (s/fALS) were K3E, D90A and L144S predicted as neutral (accuracy 63%). No statistical association was identified for results of analysis with NetDiseaseSNP. For details see Supplementary Table S3.

### Analysis of alternative splicing potential

An analysis of the possible impact of the 15 mutations on alternative splicing with three web tools (HSF 3.0, EX-SKIP, and BGBD) showed inconsistent results. HSF 3.0 showed that most of the mutations could alter splicing, while BDGE found that only S105L potentially created a new acceptor site. For details see Supplementary Table S4.

### Analysis of SOD1 protein aggregation potential

Analysis of SOD1 protein aggregation potential Aggrescan, TANGO, and Aggrescan3D 2.0 software were used to predict the tendency for aggregation of mutated protein. Aggrescan software used to predict hot spots of aggregation indicated four mutations (K3E, G38R, N139D, and L144S) as less aggregation-prone than the wild-type (WT) SOD1 sequence.

The Aggrescan and Aggrescan3D indicated nearly no mutation-induced changes in the aggregation potential for other mutations. For details see Supplementary Table S5, Supplementary File 2 and Supplementary File 3. Aggrescan3D indicate lower aggregation propensity for region of mutation D109Y in mutated dimers (D109Y/D109Y and D109Y/ref) compared to reference dimer. Tango, based on the physico-chemical principles of beta-sheet formation, extended by the assumption that the core regions of an aggregate are fully buried indicate K3E, A4V and S105L as more aggregation prone, whereas L126*, L144S as less aggregation prone comparing to WT sequence.

### Associations of clinical end-points with mutations and their molecular modeling results

Due to a limited number of available samples, the statistical analyses were available only for five mutation: K3E, L144S, G41S, L126*, and N139D.

The overall survival differed significantly between the K3E, L144S, G41S, L126* and N139D mutation carriers (p = 0.00028, chi^2^ test, Supplementary Figure S16). It was significantly shorter for G41S compared to other four mutations (p < 0.025). The survival in L144 mutation carriers was significantly longer compared to patients harboring G41S, K3E and N139D mutations (p < 0.05). Molecular predictors associated with longer survival included: neutral mutation according to PredictSNP software (p = 0.0034, Supplementary Fig. S17), neutral or mild potential consequence of mutation according to molecular modeling (p = 0.024, Supplementary Fig. S18), decrease aggregation propensity by TANGO (p = 0.012, Supplementary Fig. S19) and mutation of variable residues by Consurf (HR 0.43, 95% CI 0.23–0.81, p = 0.0086). The multivariate Cox regression model adjusted for patients’ gender and age of ALS onset showed that the deleterious mutation predicted by PredictSNP software was the strongest independent predictor of death (HR 4.47, 95% CI 1.98–10.11, p = 0.00032).

The time since the first ALS symptoms onset to respiratory insufficiency significantly differed between patients carrying K3E, L144S and G41S mutations (p = 0.00022, chi^2^ test, Supplementary Fig. S20). It was the longest for L144S (no patient reached the end-point), the shortest for G41S (all patients reached the end-point within 2 years) and medium for K3E; the differences were significant for all 3 pairs of mutations (p < 0.02). A longer time to respiratory insufficiency was associated with neutral mutation according to PredictSNP software (p = 0.0027, Supplementary Fig. S21), not altered splicing predicted with HSF 3.0 software (p = 0.0040, Supplementary Fig. S22), neutral or mild potential consequence of mutation according to molecular modeling (p = 0.0036, Supplementary Fig. S23), decrease aggregation propensity by TANGO software (p = 0.012, Supplementary Fig. S24) and mutation of variable residues by Consurf (higher value) (HR 0.28, 95% CI 0.11–0.68, p = 0.0052). The multivariate Cox regression model adjusted for patients’ gender and age of ALS onset showed that the deleterious mutation predicted by PredictSNP software was the strongest independent predictor of the development of respiratory insufficiency (HR 23.76, 95% CI 4.87–115.86, p = 0.00009).

The time since ALS onset to bulbar involvement differed significantly between the K3E, L144S and G41S mutation carriers (p = 0.0048, chi^2^ test, Supplementary Fig. S25). Harboring the L144S was associated with a significantly longer time to bulbar involvement than K3E (p = 0.0015, log-rank test) and with borderline significance longer time than G41S (p = 0.050), without significant difference between K3E and G41S (p = 0.44). The time to bulbar involvement was significantly longer in familial compared to sporadic ALS cases (p = 0.0095, Supplementary Fig. S26). The longer time to bulbar involvement was also associated with decrease aggregation propensity by TANGO software prediction when compared to neutral or increase aggregation propensities (p = 0.00043, Supplementary Fig. S27) and with potentially not altered splicing when compared to potentially altered splicing according to HSF 3.0 (p = 0.00062, Supplementary Fig. S28).

Time since ALS onset to a wheelchair use was significantly longer for L144S compared to K3E (p = 0.0039, Supplementary Fig. S29). A longer time to wheelchair use was also associated with a decrease aggregation propensity by TANGO software prediction, when compared to neutral or increase aggregation propensities (p = 0.0031, Supplementary Fig. S30).

We have found that a more advanced age at disease onset was linked to a shorter survival (HR 1.046) with a growing risk of death of 4.6% at every additional year at onset. Further association analysis of clinical data revealed that the age of ALS onset was significantly lower in L144S compared to patients carrying the K3E mutation (45.8 ± 9.8 vs 53.3 ± 8.1 years, p = 0.019). The G41S was associated with upper limbs onset as compared to other frequent mutations (K3E, L144S, L126* and N139D, p = 0.017). The PMA phenotype was significantly more frequent among the N139D mutation carriers as compared to K3E (100% vs 19%, p = 0.021).

## Discussion

From over 185 *SOD1* mutations identified to date, only some (e.g., H46R, D90A, and R115G) cause ALS with a defined clinical phenotype, including characteristic age of onset, survival time and/or site of onset (lower limbs in D90A and H46R). Other mutations present a more varied course or have only been identified in individual patients/families, making a comprehensive analysis of the genotype–phenotype relation difficult.

The frequency of *SOD1* mutations in fALS patients varies between populations from 13 to 20% and in sALS patients from 1 to 2%^28–30^. Thus the mutation frequency in the present study (21.1% in fALS, 2.3% in sALS) is relatively high. The L144S and K3E variants were the most frequent among the Polish ALS patients (but not in other populations, except for L144S among Brazilian patients^31–33^. Contrary, D90A, the most frequent European *SOD1* mutation, was only present in 0.4% of cases (4.1% of fALS). From the mutations with the highest number of representing individuals the L144S was associated with the earliest disease onset and the slowest progression, while the G41S was characterized by a particularly aggressive progression and a short survival, comparable with the phenotype reported for A4V in North American population^34^. Although the number of affected individuals were not sufficient for statistical analysis, based on the clinical observation, the individuals with G37R, N86S, homozygous D90A or G93C mutations presented with a relatively less severe, while A4V, G72S, and S105L with more severe phenotypes (based on time for reaching clinical end-points), similarly to previous reports from other populations^9,35–38^. The L126* mutation, previously described as aggressive, showed a highly variable survival in our study, ranging from 36 to 228 months^39^. As for the clinical phenotypes, the statistical analysis showed that N139D mutation was linked to the most homogenous clinical presentation of PMA, as opposed to the K3E characterized by the prevalence of classic ALS. From the clinical observations: beside the N139 D, the isolated lower motor neuron involvement (PMA) was observed in the G72S, N86S, G93C, S105L and L126*, while the classic phenotype prevailed among patients with the K3E, G41S, D90A, and L144S mutations, and was also shown in A4V, W32* and G37R represented by single individuals. The D90A homozygotes shared the classic phenotype with prevalent lower motor neuron involvement and lower limbs onset, whereas one of the patients heterozygous for D90A had a bulbar onset and a short survival of 25 months. We cannot exclude that the MSA-P in the patient with the D109Y mutation was an accompanying condition, as the mutation had previously been described in classic ALS with prevalent UMN involvement, bulbar onset and long survival^40^. In contrast to other studies we found a highly infrequent onset in the upper limbs among patients harboring *SOD1* mutations.

All the cds mutations identified in our study group were classed as disease-causing according to the *Human SOD1 non synonymous SNP analysis* (http://bioinfogroup.com/sod1/snp/), which is consistent with our prioritization results^41^. However, according to the *SNP analysis* database none of the mutations was predicted to influence the aggregation tendency or amyloid propensity. Our protein modeling results (Table 3, Supplementary Figs. S1–S15) in which we compared WT and mutated SOD1 structures after the all-atom MD simulations of a SOD1 dimer in a water environment imitating mammalian cytosol conditions^42^ showed that only two mutations, D109Y and C111Y, had an increased aggregation potential. However, these mutations were not considered aggregation-prone by SNP analysis, Aggrescan, TANGO or Aggrescan3d (Supplementary Table S5). This is concordant with previous observations that SOD1 in fALS has a reduced propensity to form aggregates, while soluble heterodimers and trimeric SOD1 complexes may be more toxic as compared to large aggregates^43,44^.

A slightly higher aggregation potential was predicted for the A4V mutant. The movement of loops adjacent to residue after the A > V mutation is considerable (Supplementary Fig. S3), which can facilitate aggregation. After the mutation, the valine side chain is directed towards the center of the b-barrel rather than exposed but it still could promote movements of adjacent fragments. Ours result appears to support the hypothesis that folding intermediates of SOD1 are an important source of cytotoxic conformations in ALS pathology^6^. Indeed, it was previously proposed that A4V mutation destabilizes SOD1 monomer and weaken the dimer interface^45^. Recently Brasil et al. observed low levels of SOD1 monomers in cells co-expressing WT and A4V SOD1, and the predominant formation of heteromeric species^46^. Based on this they suggested that WT SOD1 might exist primarily as unfolded monomeric intermediates and then fully active dimers. On the other hand, unfolded and misfolded monomers might be the predominant mutant SOD1 form.

We found L126* to be the most damaging to the protein structure. The truncation of a substantial fragment of the polypeptide (loop 126–141 and β-thread 142–151) rearranges both the dimer interface and the active site. The reported differences between the results concerning SOD1 protein stability obtained with different methods (bioinformatics, molecular modeling, in vitro, in vivo) suggest the existence of as yet unidentified factors involved in the formation of pathogenic SOD1 conformations in vivo^43,47^. *SOD1* gene variants undergoing alternative splicing have already been described in fALS patients^48,49^. In the present study potential for alternative splicing due to the cds mutations was disputable, as only some of programs used indicated a small probability of such an effect. Nevertheless, the effects of two of those mutations, W32* and S105L seems sufficiently likely to deserve an experimental verification. Potentially altered splicing predicted with HSF 3.0 software was significantly associated with bulbar involvemenhuman spt in sALS patients and respiratory insufficiency in familial and sporadic ALS patients.

We observed that the least evolutionarily conserved positions in SOD1 (D90 and L144) were also the most frequently mutated in our study group, and the D90A and L144S carriers suffered from a slowly progressing ALS. Mutations of conserved residues of SOD1 were significantly associated with shorter survival times and shorter time between the disease onset and respiratory failure in ALS patients. Similarly, the PredictSNP (a consensus classifier for prediction of disease-related amino acid mutations) classified the K3E, D90A, D109Y, and L144S mutations as neutral and their carriers’ symptoms were relatively less severe in terms of age of onset and survival times.

The pathogenicity of at least some ALS-related SOD1 mutations seems to involve the formation of amyloid-like aggregates. However, Aggrescan classified WT SOD1 and nearly all its mutated versions studied here as of low aggregation propensity (highly negative Na4vSS scores); still, specific nucleation points from which an ordered fibrillary structure could spread under certain conditions would nevertheless make the mutated protein amyloidogenic. Notably, the K3E, L144S, and N139D variants were predicted to be even less aggregate-prone than the WT SOD1, while A4V was the only mutant with a markedly enhanced aggregation potential. Algorithms that have been derived from and used to predict amyloid fibril formation in the absence of other biological factors also offer a considerable degree of accuracy for predicting amyloid-aggregation propensity in vivo remains to be improve to extend this prediction to disease manifestation and pathology^50^. Statistical analyses indicate some ALS clinically relevant end-points (bulbar involvement, wheelchair-bound, respiratory insufficiency, survival time) with increase aggregation propensity but also neutral by TANGO software prediction. To sum up, while the prediction programs and molecular modeling could not define consistently the pathogenicity of each and every *SOD1* mutation, there was a good agreement between those predictions and the disease severity for the both ends of the ALS spectrum, i.e., the least and the most severe cases.

A caveat of our study is that the only experimentally-derived SOD1 structure available is a dimer of the mature molecule, whereas the prion-like ALS pathomechanism is most probably associated with an immature or misfolded protein^6^. For instance it was shown recently that both WT and mutant SOD1 form dimers and oligomers, but only mutant SOD1 aggregates and form intracellular inclusions. Moreover, co-expression of WT and mutant SOD1 in various cell models resulted in the formation of a larger number of inclusions, as compared to cells expressing WT or mutated SOD1 separately^51^. Taking into consideration the dysfunction of numerous cellular pathways observed in ALS, aggregation of SOD1 does not seem to be the only cause of ALS. According to the multistep hypothesis of ALS^52^, single *SOD1* mutations may influence more than one step leading to ALS onset.

The major effect of *SOD1* mutations in ALS is linked to the protein aggregation and a prion-like propagation of misfolded molecules. These mutations may also lead to a loss of function of SOD1 by affecting its structure and/or interactions pattern. The loss of function involves not only the dismutase enzymatic activity, e.g., associated with the N86S mutation^53^, but may also involve a loss of the nuclear function where SOD1 acts as a transcription factor^54^. In one sporadic ALS patient we identified a nonsense mutation at codon 32 (p.W32*), which was absent from the whole exome/genome databases (1000 GP, gnomAD)^55^. Since the W32* was also found in the patient’s asymptomatic mother, age > 70, we were not able to prove the mutation was pathogenic. We further found that SOD1 W32* was associated with a dismutation activity in erythrocytes reduced by half^53^, which might point at the loss of SOD1 function^56,57^. The premature stop codon could result in the shortest reported truncated SOD1 protein, but most likely the nonsense-mediated mRNA decay prevents the synthesis of such abnormal protein^58^.

A putative loss of SOD1 function in ALS was reported in previous studies. For instance, the mutation V30Dfs*8^59^ should produce a very short, non-functional truncated SOD1 protein. G28_P29del caused by alternative splicing of exon 2 of *SOD1* leads to reduced transcription and a low level of SOD1 protein in the mutation carriers^60^. A similar result was reported for mutation S108Lfs*15^61^, as the authors observed ca. 50% reduction of the SOD1 protein level and could not detect the truncated SOD1 (a protein with the predicted molecular weight) by Western blotting. The above mentioned *SOD1* mutations, as well as other pathogenic variants including D90A, G41S, and I112M^62,63^, showed a reduced penetrance.

Interestingly, a recent in vitro study has shown that the tryptophan residue at position 32 (W32) is necessary for the formation of a competent seed for aggregation allowing the prion-like propagation of SOD1 misfolding from cell to cell, and the W32S substitution blocked this phenomenon^64^. Also a study on SOD1 single copy/knock-in models of ALS in *C. elegans* suggests an involvement of both the loss and gain of function of SOD1 in ALS development^65^. The contribution of the loss and gain of function mechanisms vary in different neuronal populations. In the studied model, a glutamatergic neuron degeneration was induced by oxidative stress due to the loss of SOD1 function, a phenomenon also observed in a significant fraction of ALS patients. Also recent reports on children with a homozygous truncation mutation (p.C112Wfs*11) with no SOD1 activity and severe symptoms during infancy suggest that the loss of SOD1 enzymatic activity contributes to motor neuron disorders^66,67^.

To sum up, the SOD1 haploinsufficiency with all its consequences might be one of the factors in an oligogenic etiology of ALS. It is most likely that many cases of ALS are due to the presence of multiple gene variants with different pathogenicity. Understanding the input of such variants to the development of neurodegeneration and their interactions with diverse environmental factors (e.g., toxins or the microbiome) is critical for the development of efficient therapies, especially in regards to potential gene therapy^4,68^.

## Conclusions

We found L144S and K3E to be the most frequent SOD1 mutations among Polish ALS patients. Carrying L144S mutation was linked to the longest, while G41S to the shortest overall survival. Despite intrafamilial heterogeneity, L144S was significantly associated with the least severe, K3E with medium and the G41S the most aggressive disease progression. In silico analysis and molecular modeling of SOD1 variants allowed to identify relationsbetween *SOD1* mutations and the ALS clinical phenotype. The time to the clinically relevant endpoints including walking loss, bulbar involvement, respiratory insufficiency and overall survival, were significantly associated with the in silico predictions results including substantial disturbance of SOD1 structure. However, our analyses indicate that the in silico prediction of mutation consequences might be incompatible with disease course (i.e. N86S-less severe phenotype and severe mutation consequences predicted by used software).

## Supplementary Information

Supplementary Information 1.

Supplementary Information 2.

Supplementary Information 3.
