# Supplementary material for: SOD1 mutations associated with amyotrophic lateral sclerosis analysis of variant severity
Source: Sci Rep. 2022 Jan 7;12:103. doi: 10.1038/s41598-021-03891-8 (PMC8742055; doi:10.1038/s41598-021-03891-8)
Supplement: Supplementary file 3 — Supplementary Information 3. [file 41598_2021_3891_MOESM3_ESM.pdf]

SOD1 mutations associated with amyotrophic lateral sclerosis analysis of variant severity

Mariusz Berdyński[1,2&*], Przemysław Miszta[3&], Krzysztof Safranow[4&],Peter M. Andersen[2], Mitsuya Morita[5] , Sławomir Filipek[3], Cezary Żekanowski[1], Magdalena Kuźma-Kozakiewicz[6,7*]

[1] Laboratory of Neurogenetics, Department of Neurodegenerative Disorders, Mossakowski Medical Research Institute, Polish Academy of Sciences, Warsaw, Poland

[2] Department of Clinical Sciences, Neurosciences, Umeå University, Sweden

[3] Faculty of Chemistry, Biological and Chemical Research Centre, University of Warsaw, Poland.

[4] Department of Biochemistry and Medical Chemistry, Pomeranian Medical University, 72 Powstańców Wlkp. Str., 70-111 Szczecin, Poland

[5] Division of Neurology, Department of Internal Medicine, Jichi Medical University, Shomotsuke, Japan

[6] Department of Neurology, Medical University of Warsaw, Poland

[7] Neurodegenerative Diseases Research Group, Medical University of Warsaw, Poland

[&]these authors contributed equally to this work

*Corresponding authors:

Magdalena Kuźma-Kozakiewicz MD, PhD
E-mail: mkuzma@wum.edu.pl

Mariusz Berdyński PhD
e-mail: m.berdynski@imdik.pan.pl

Supplementary Information

Supplementary figures:

Supplementary Figures S1-S15. Structural Figure: K3E, A4V, G37R, G41S, G72S, N86S, D90A, G93C, S105L, D109Y, C111Y, L126*, N139D and L144S.

Supplementary Figures S16-S30. Statistical analysis

Supplementary tables:

Supplementary Table S1. ConSurf results of mutated aminoacids in SOD1

Supplementary Table S2 PredictSNP and NetDiseaseSNP results

Supplementary Table S3 Alternative splicing analysis by Human Splice Finder, Ex-Skip, BDGP splite Site Prediction

Supplementary Table S4 Aggrescan analysis (ttp://bioinf.uab.cat/aggrescan

Supplementary figure S1-S15. Structural Figures. Comparison of SOD1 structures: wild type and mutants. WT structure (carbon atoms) is colored in gray but the residue which will be mutated in purple. For mutated protein the carbon atoms are colored in cyan but carbon atoms of mutated residue are colored in yellow. The region of mutation is encircled.

Figure S1. Mutation K3E.

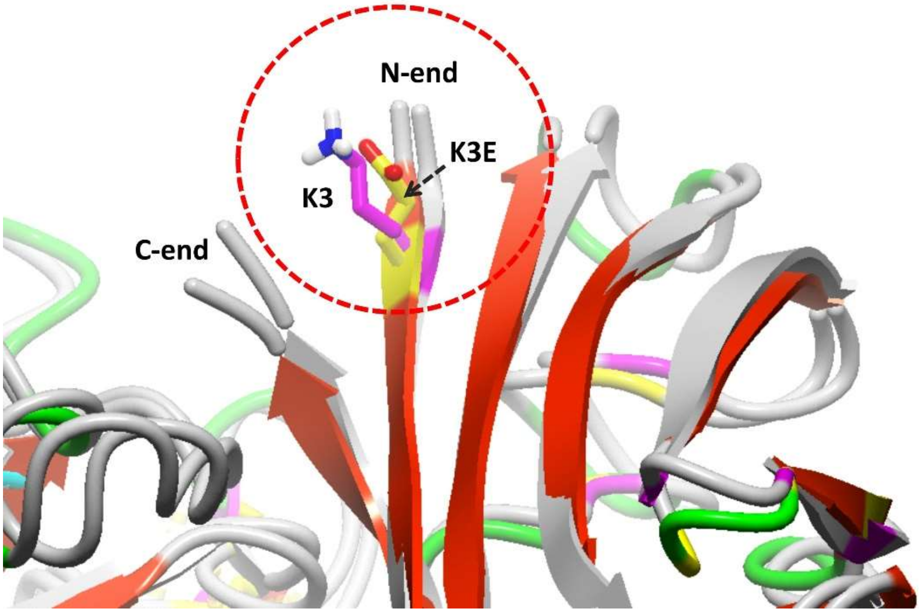

Figure S2. Mutation K3E. Comparison of electrostatic potentials mapped on molecular surfaces of of SOD1 dimers. Colors: red – negative potential, blue – positive potential.

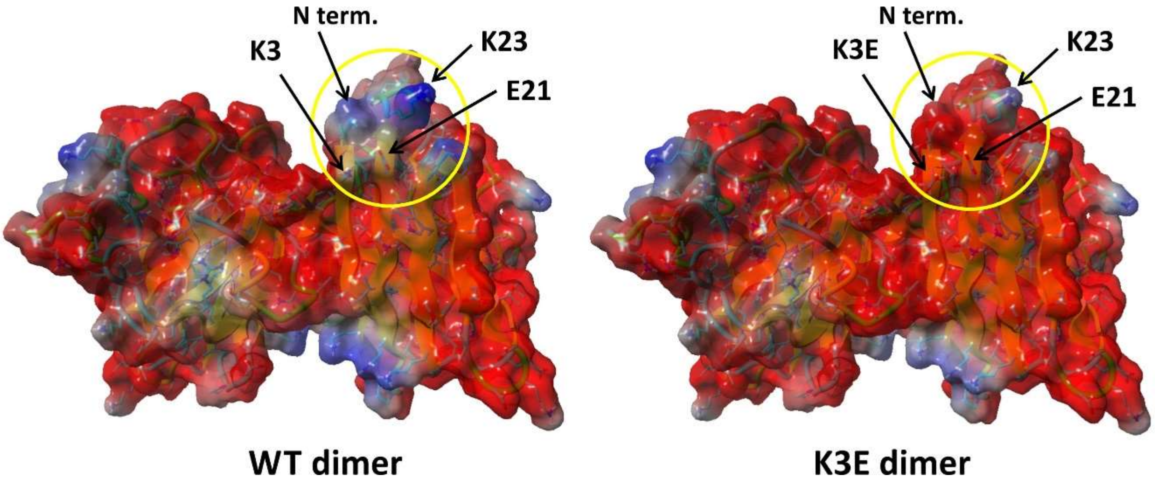

Figures S3. Mutation A4V. Red arrows indicate movements of residues and loops.

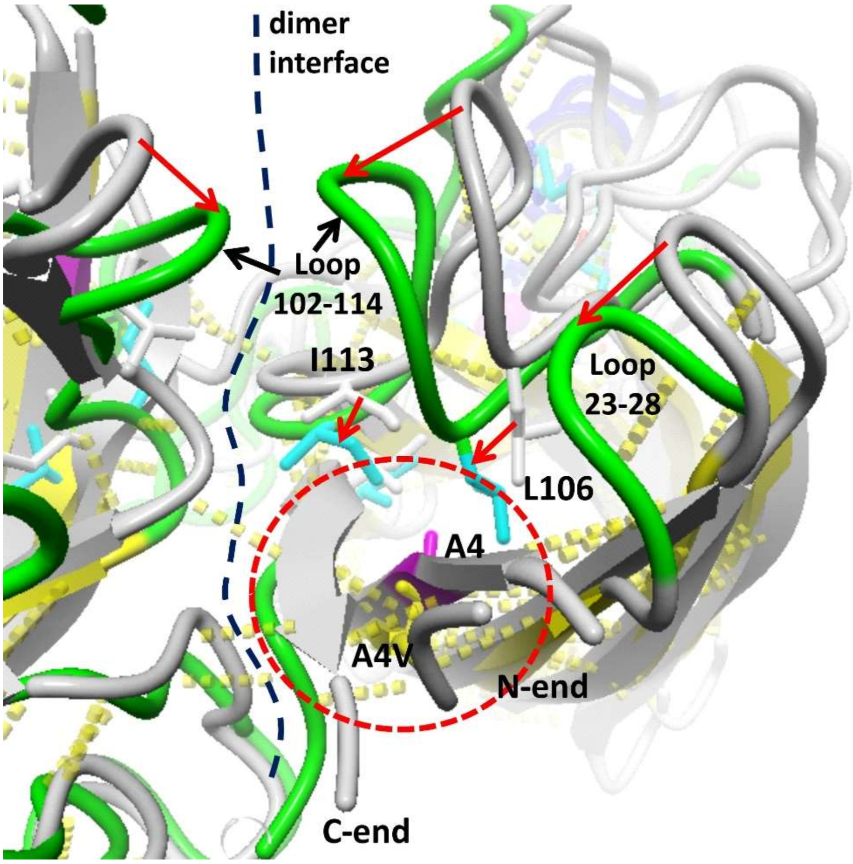

Figure S4. Mutation G37R

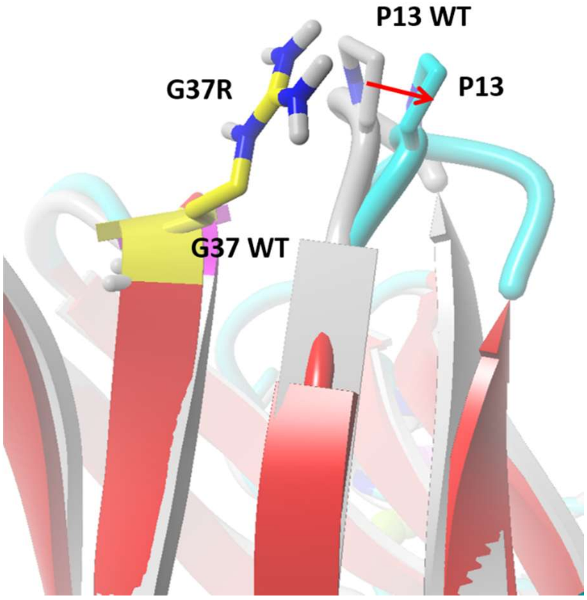

Figure S5. Mutation G41S.

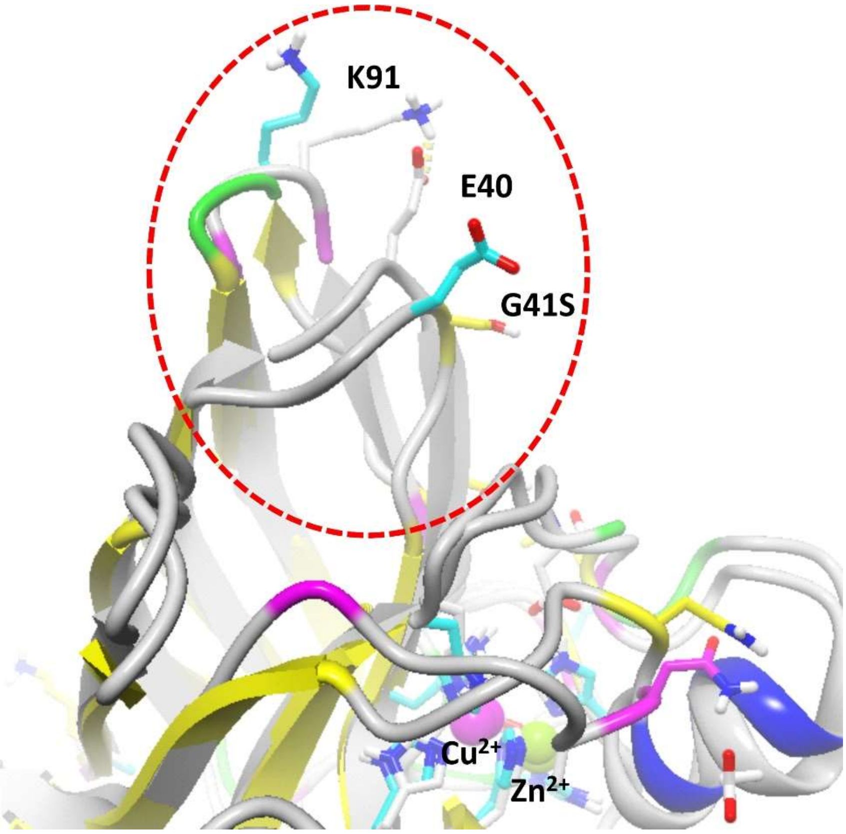

Figure S6. Mutation G72S.

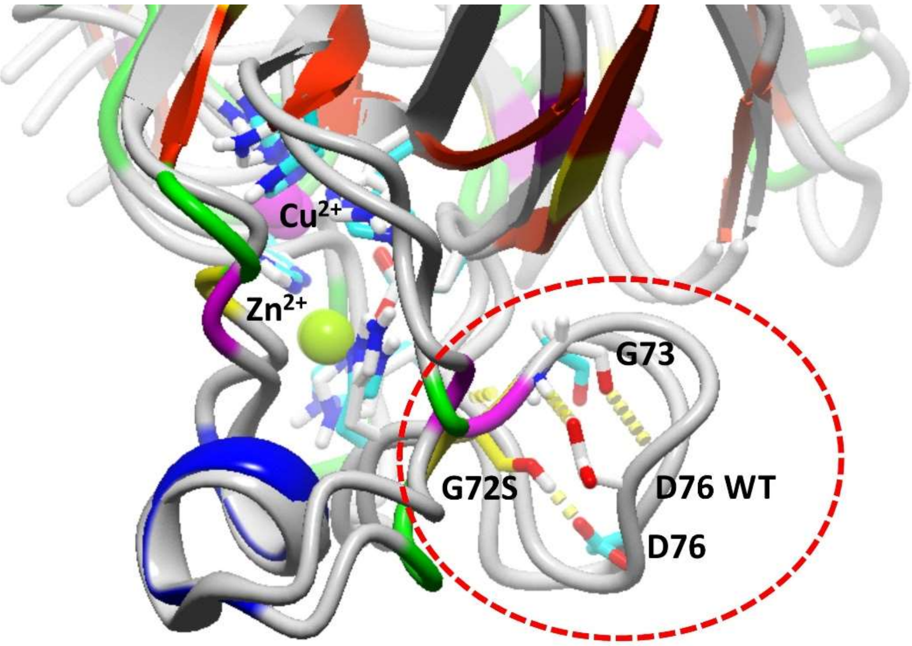

Figure S7. Mutation N86S.

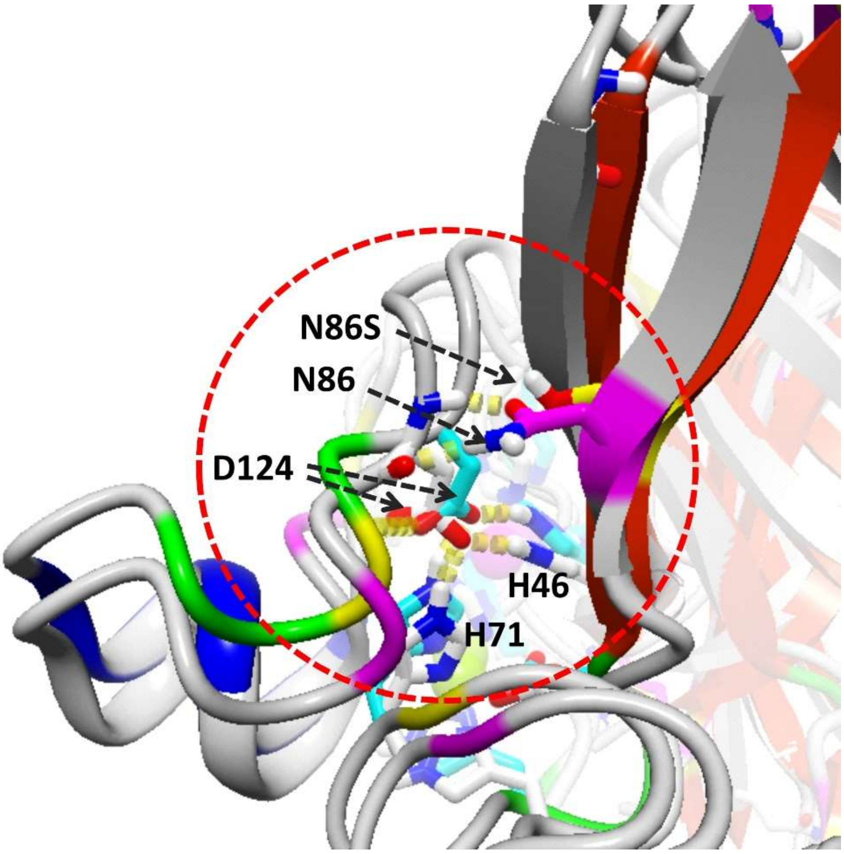

Figure S8. Mutation D90A.

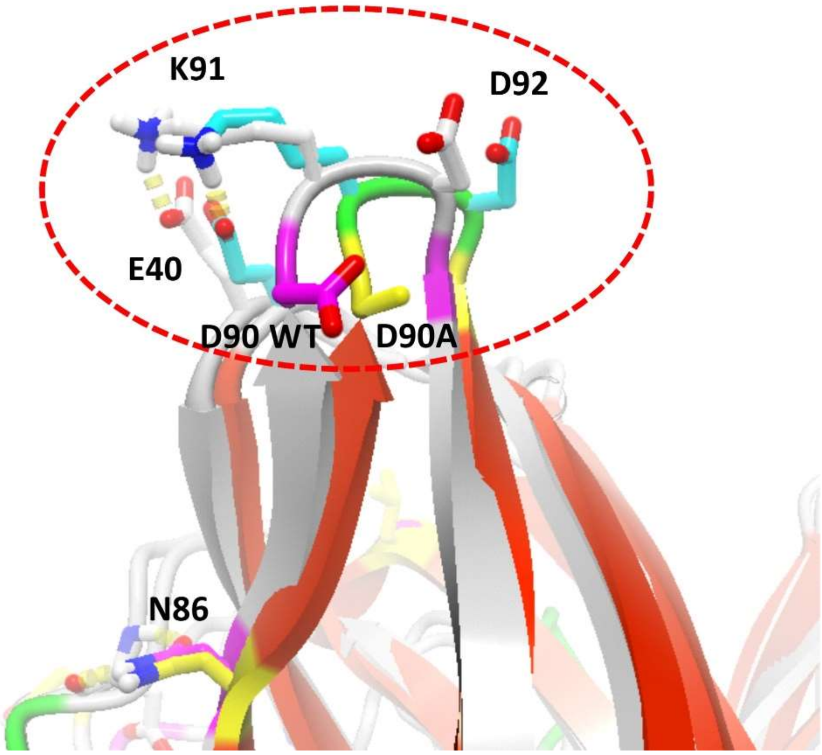

Figure S9. Mutation G93C.

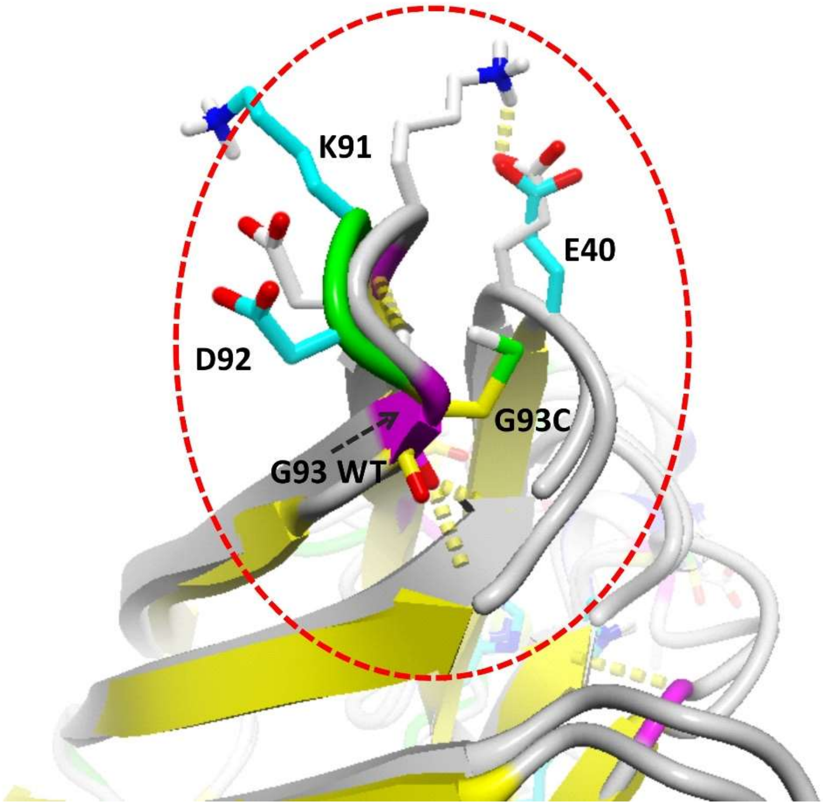

Figure S10. Mutation S105L

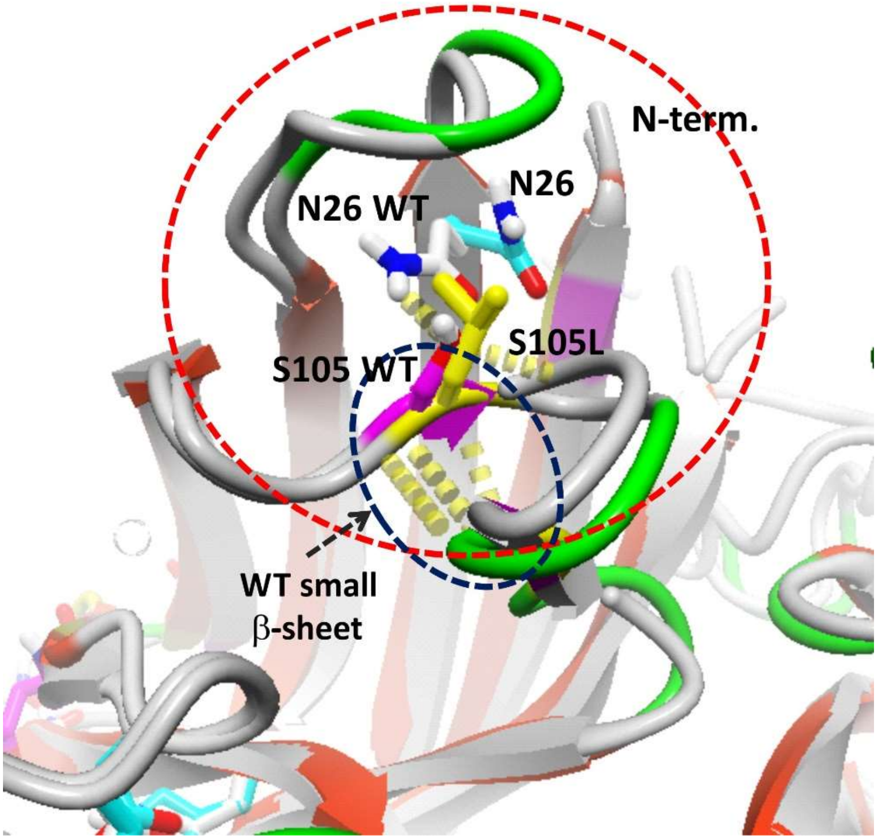

Figure S11. Mutation D109Y.

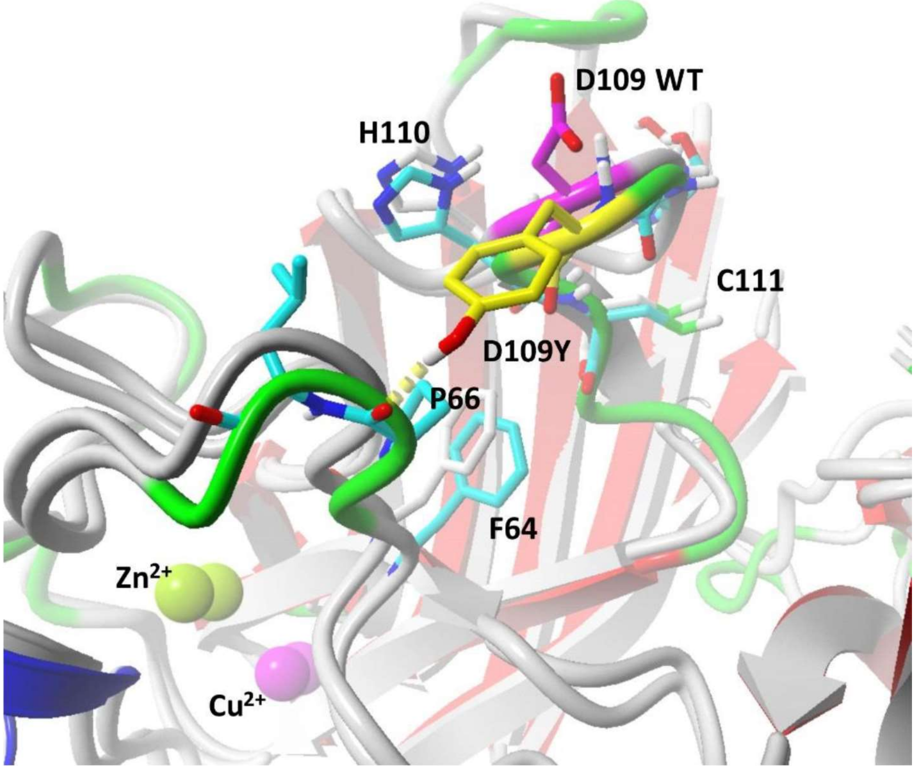

Figure S12. Mutation C111Y.

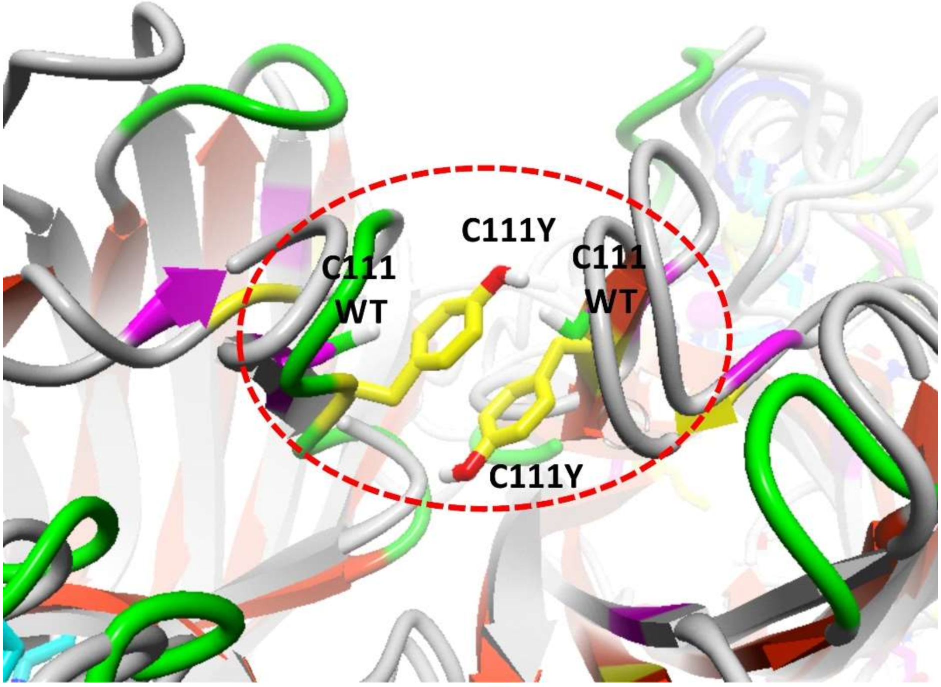

Figure S13. Mutation L126*.

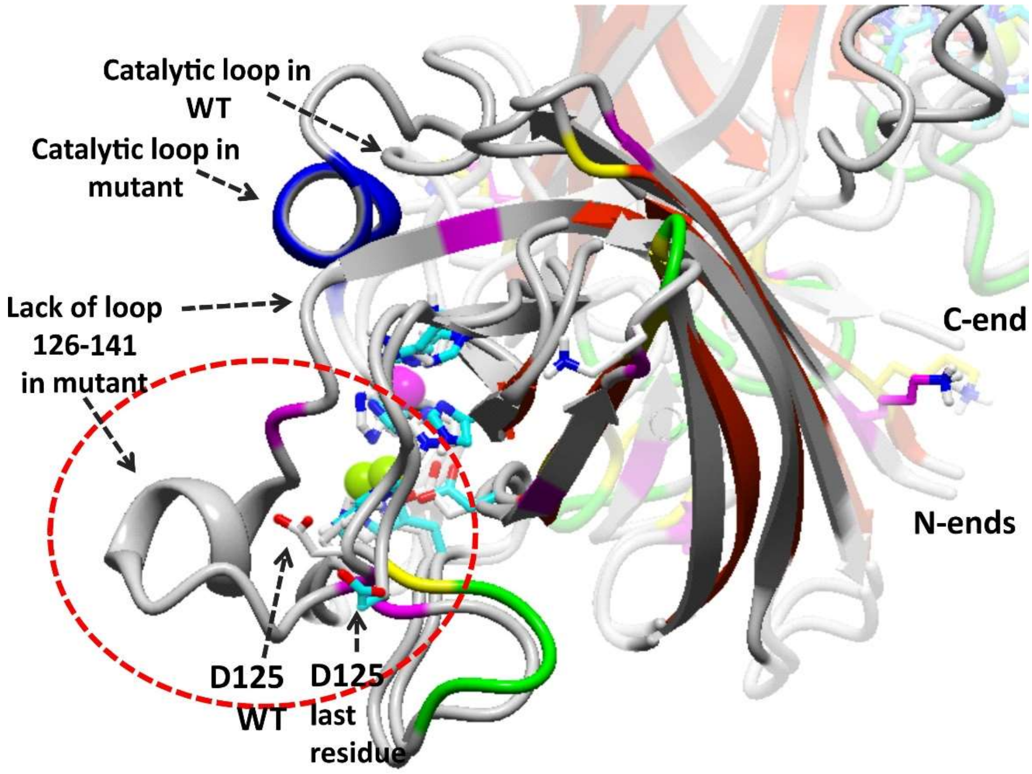

Figure S14. Mutation N139D.

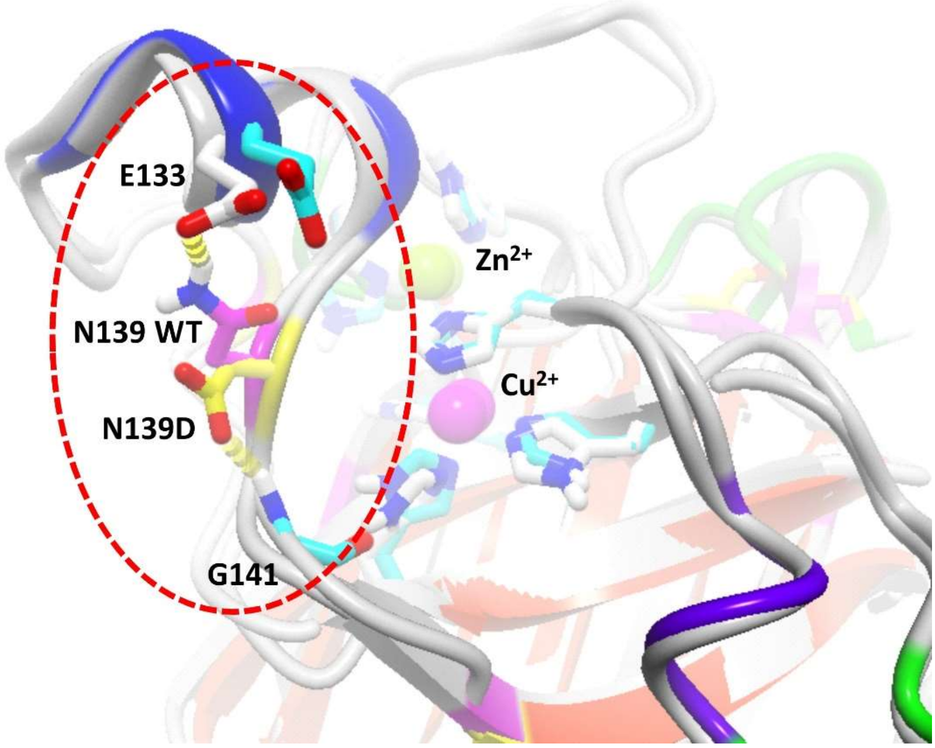

E133
Zn^{2+}
N139 WT
Cu^{2+}
N139D
G141

Figure S15. Mutation L144S. Red arrows indicate movement of residues of one end of β-sandwich after mutation.

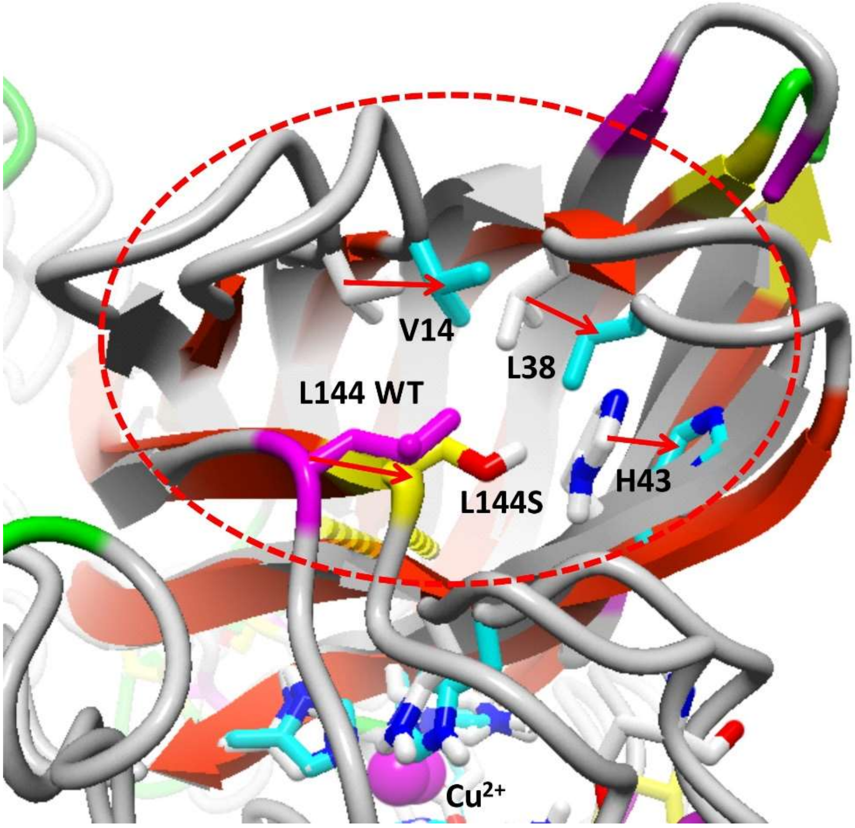

K3E
This mutation does not change the protein structure. The β-thread, in which K3E is located, preserves all hydrogen bonds with adjacent β-threads of the same β-sheet (Figure 1). What is changed is the external electrostatic potential. The SOD1 dimer contains 42 negatively charged residues and 30 positively charged residues. The K3E mutation additionally increases the negative charge of protein, and although it is not a large change globally, at a local scale it neutralizes a positive charge from adjacent N-terminus (Figure 2) and causes a radical change on the electrostatic potential of this region from +2 to zero and repulses the adjacent C-terminus.

A4V
Mutation A4V leads to large movements of loops located close to the area of the mutation and in the dimer interface (Figure 3). Movements of residues L106 and I113 were about 2 Å, movement of loop 23-28 about 6 Å, while two loops 102-114 located symmetrically on both sides of the interface moved 5-6 Å towards one another but did not form any interaction during 20 ns MD simulation. Parts of β-threads in a mutation area from N- and C-termini, comprising of residues 2-3 and 150-152, were dissolved. Mutation A4V also makes a global movement: there is a rotation of the whole β-sandwich domain in both monomers about 12° so the interface is greatly modified what can preclude binding of other proteins to the groove between monomers.

G41S

G41 is located at the end of a short loop between two β-threads (Figure 4). This mutation dissolves slightly the β-thread it is located in (residues 41-43). A salt bridge located nearby at E40-K91 is broken, however, in the second monomer of SOD1 it remains unchanged and indicates a large flexibility of this loop prolonged by three residues. Serine G41S is directed outside of protein and does not form any internal interactions. Thus, this mutation is likely to influence interactions with other protein and a potential formation of hydrogen bonds. In this case the interaction strength would be increased, or vice versa, the increased dimensions of G41S residue could preclude binding.

N86S

The residue N86 in WT forms a hydrogen bond with N-H group of D124 residue (Figure 5). A side chain of D124 forms hydrogen bonds and stabilizes two histidine residues; H71 that coordinates Zn^{2+} and H46 that coordinates Cu^{2+}. In the N86S mutant the new serine residue does not interact with D124. The residue D124 is located in a flexible loop so the lack of stabilization of this residue might perturb binding of metal ions and lead to decreased SOD1 activity.

D90A

The residue D90 is located at the beginning of a short loop between two β-threads (Figure 6). D90 does not interact with adjacent lysine K91, which forms a salt bridge with E40, so the mutation does not break this bridge. The protein structure is also not changed. The only modification is a drastic change of electrostatic potential of this highly charged, short loop DKDG to AKDG that changes its negative charge to neutral. This change could prevent binding of other proteins or, conversely, some other protein can bind employing the modified electrostatic potential of this site.

G93C

Residue G93 is located at the end of short loop 90-93 DKDG (Figure 7). Mutation G93C dissolves slightly part of β-thread (residues 93-94) and also the adjacent β-thread is slightly dissolved (residue 38). Due to increased flexibility of both loops the salt bridge E40-K91 is broken. Not only an increased flexibility of these loops but also the larger volume of mutated residue G93C could contribute to lack of matching interactions with other proteins.

S105L

Residue S105 is located in a long loop 102-115 between two β-threads (Figure 8). The position of this loop is stabilized by a breaks hydrogen bond between side chains of S105 and N26 located at the adjacent β-sheet. Mutation S105L this bond and the loop becomes more flexible. Also a change of residue character from polar to hydrophobic could destabilize interactions with other proteins. The internal hydrogen bonds in this loop in which S105L participates, are also weakened. A small β-sheet existing in this loop is lost so that the whole loop is much more flexible.

D109Y

The mutation D109Y diametrically changes the electrostatic potential and interactions of the loop 102-115 (Figure 9). D109 is located at the tip of this loop and, therefore, may serve as an orientation point for the other proteins to take the proper orientation before binding. Mutation D109Y removes the only negative charge in this loop and makes this loop neutral what completely changes potential interactions with other proteins. Furthermore, D109Y forms a hydrogen bond with residue P66, which is located in a loop 49-84 encompassing the binding site for metal ions. In WT protein these two loops are not connected so the mutation

D109Y makes both loops more rigid which can influence binding of substrate and slow down the enzymatic reaction. Making the tip of the loop 102-115 more hydrophobic increases also the possibility for oligomerization of this protein.

C111Y
Residue 111 is located in the same loop as the previously described residue 105 (Figure 10). Swapping of a cysteine to a tyrosine at position 111 leads to a change of the interface between both monomers of SOD1 so the mutation C111Y could influence the binding of other proteins in the groove between the monomers. Insertion of such a large residue changes the shape of this interface. Moreover, two tyrosine residues in 111 positions form π-π stacking interaction (*face to face*) what additionally diminishes depth of a groove between two monomers. The proteins recognizing the shape of the groove could be disturbed.

*L126**
Lack of the last part of SOD1 sequence, comprising of the loop 126-141 and the β-thread 142-151, leads to large distortions of this part of the protein structure. Long catalytic loop 49-83 containing residues that bind metal ions changes its shape in 49-62 region and an additional single α-helix turn is formed (Figure 11). The reason of that is the lack of the last part of protein sequence and especially the last β-thread so that the long loop can extend itself in this direction. This modification destabilizes the catalytic site since metal ions are bound less firmly due to increase of loop flexibility. The last but one β-thread of SOD1, comprising of residues 115-120, is disordered in L126* mutant so that it does not form a hydrogen bond with the rest of β-sheet. This β-thread contains residue H120, which binds to copper ions so disorganization of this region additionally destabilizes the active site.

N139D
Mutation N139D located in close vicinity of the catalytic site, in short loop 138-142, can influence binding of substrates especially that the electrostatic potential is changed and is more negative in this mutant (Figure 12). The pattern of hydrogen bonds is also changed: the side chain of N139 in WT forms a hydrogen bond with side chain of E133, while mutated N139D binds to main chain of G141. Lack of a connection between N139D and E133, located in a short α-helix, destabilizes this area and influences directly the active site and possibly binding of substrates.

L144S
Residue 144 is located on the surface of SOD1 far from a dimer interface. A vicinity of this residue is hydrophobic – it is one end of so called β-sandwich composed of two parallel β-sheets. After mutation L144S the hydrophilic serine residue in a hydrophobic environment and via increased interactions with water molecules, induces a collective movement of the whole end of the β-sandwich (Figure 13). The other end of the β-sandwich is moved in the opposite direction so the catalytic site located in the middle of the outer side of β-sandwich remains relatively unchanged. The β-sandwich domain in the second monomer of SOD1 dimer is moved in the opposite direction so the shape of dimer interface if modified. Thus, interactions with other proteins binding to this region could be weakened.

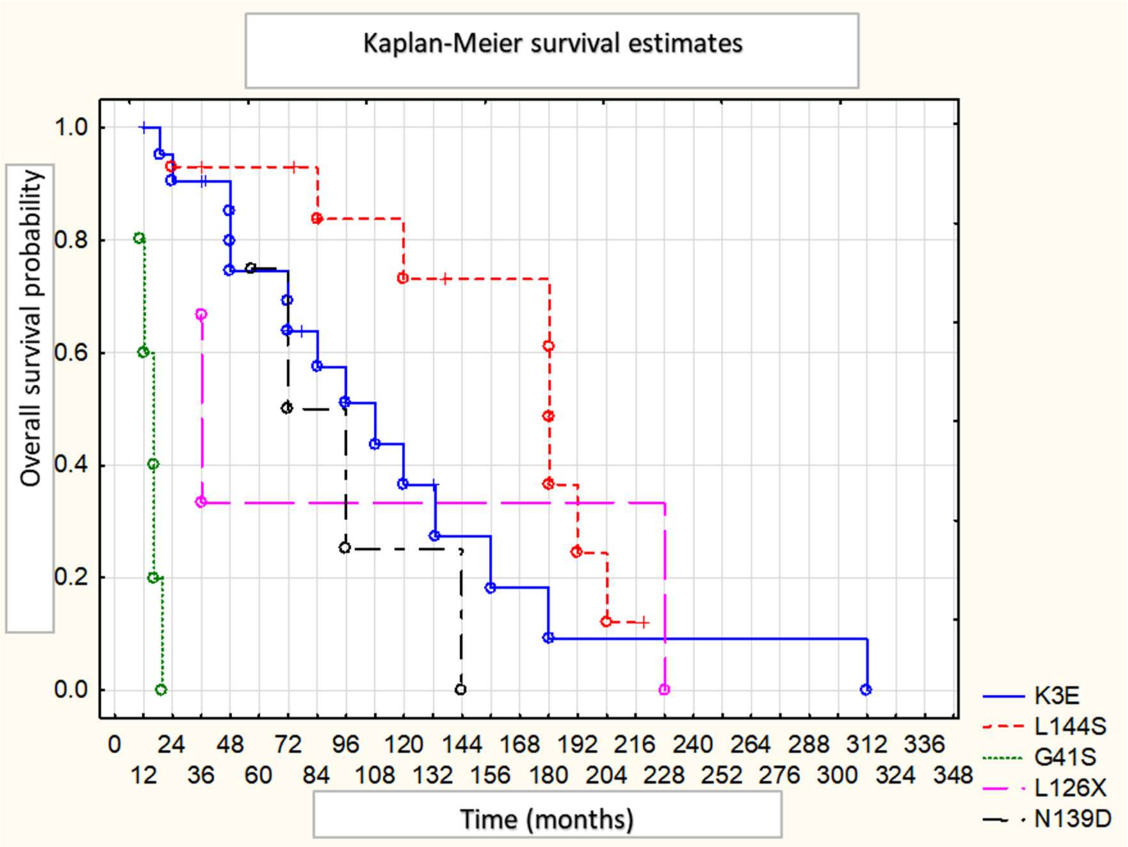

Figure S16. Comparison of overall survival between carriers of specific SOD1 mutations.
Chi2 = 21.2864 df = 4 p = .00028

K3E	vs	G41S	Log-rank test: -2.71047 p = .00672
L144S	vs	G41S	Log-rank test: -2.86806 p = .00413
L126*	vs	G41S	Log-rank test: -2.33052 p = .01978
N139D	vs	G41S	Log-rank test: -2.25363 p = .02422

K3E	vs	L144S	Log-rank test: 1.979087 p = .04781
N139D	vs	L144S	Log-rank test: -2.08115 p = .03742
L126*	vs	L144S	Log-rank test: -.216280 p = .82877

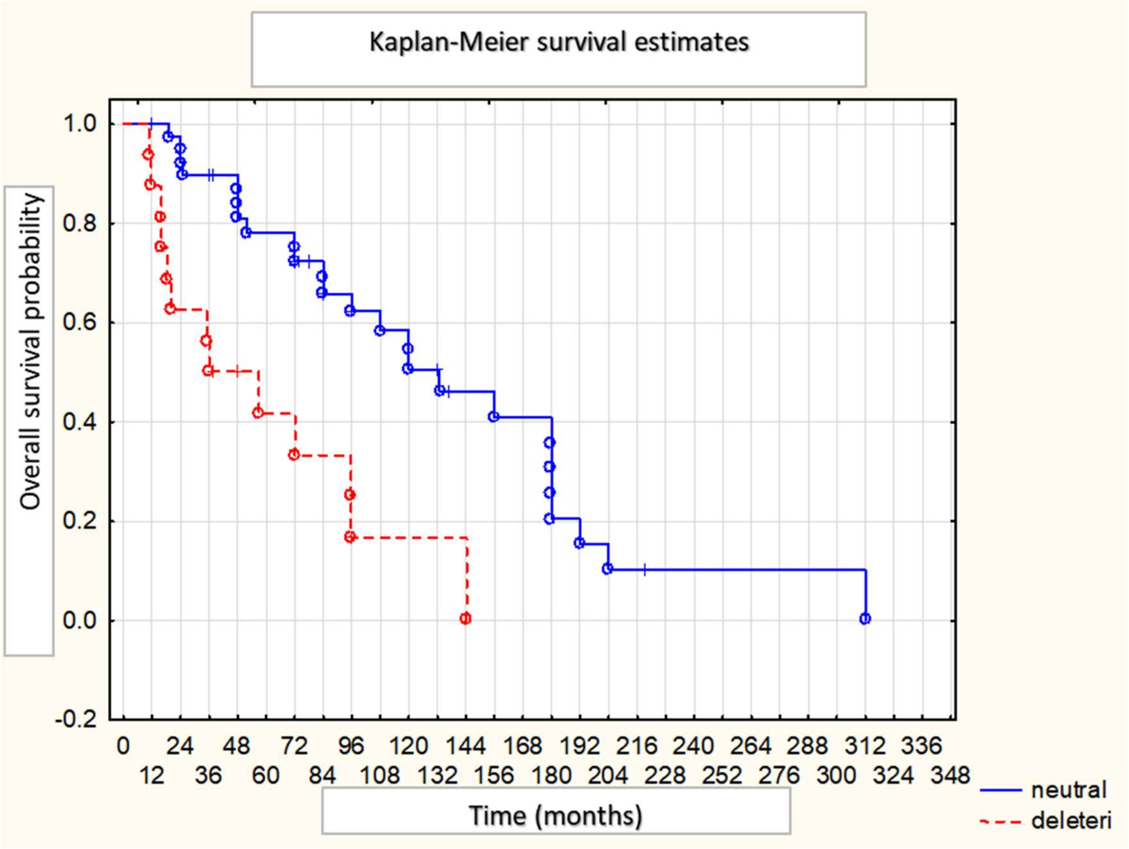

Figure S17. Comparison of overall survival between patients with neutral and deleterious PredictSNP results.

Log-rank test -2.92906 p = .00340

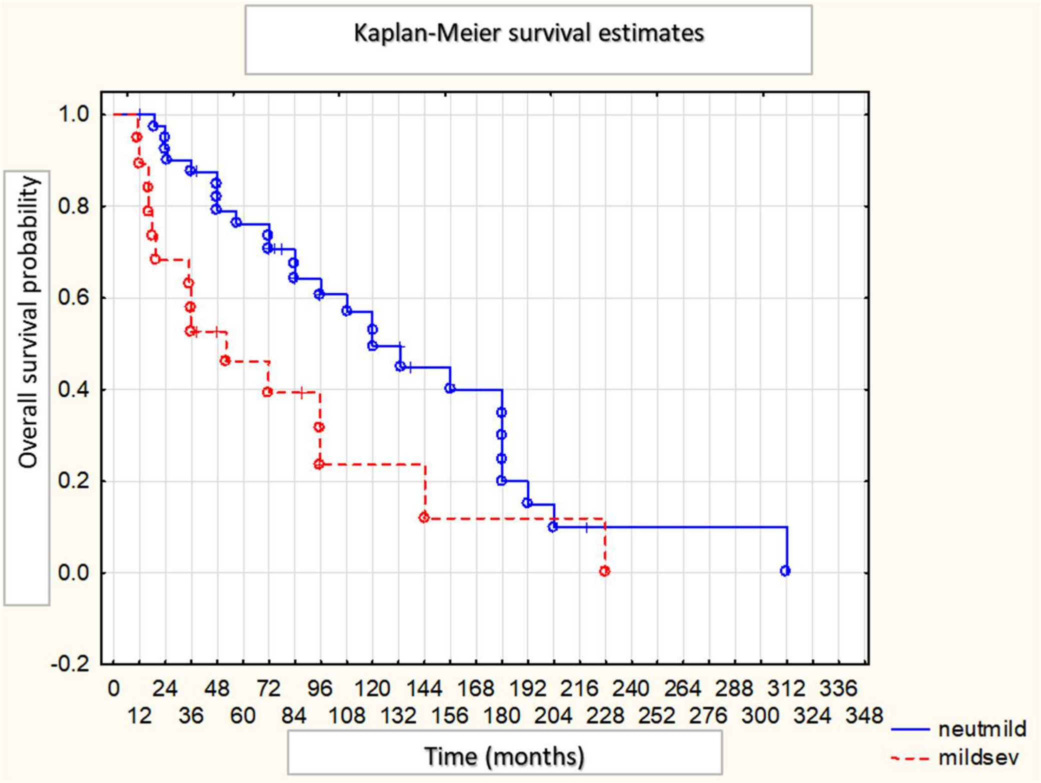

Figure S18. Comparison of overall survival between patients with neutral to mild and more severe than mild molecular modeling results.
Log-rank test -2.25163 p = .02435

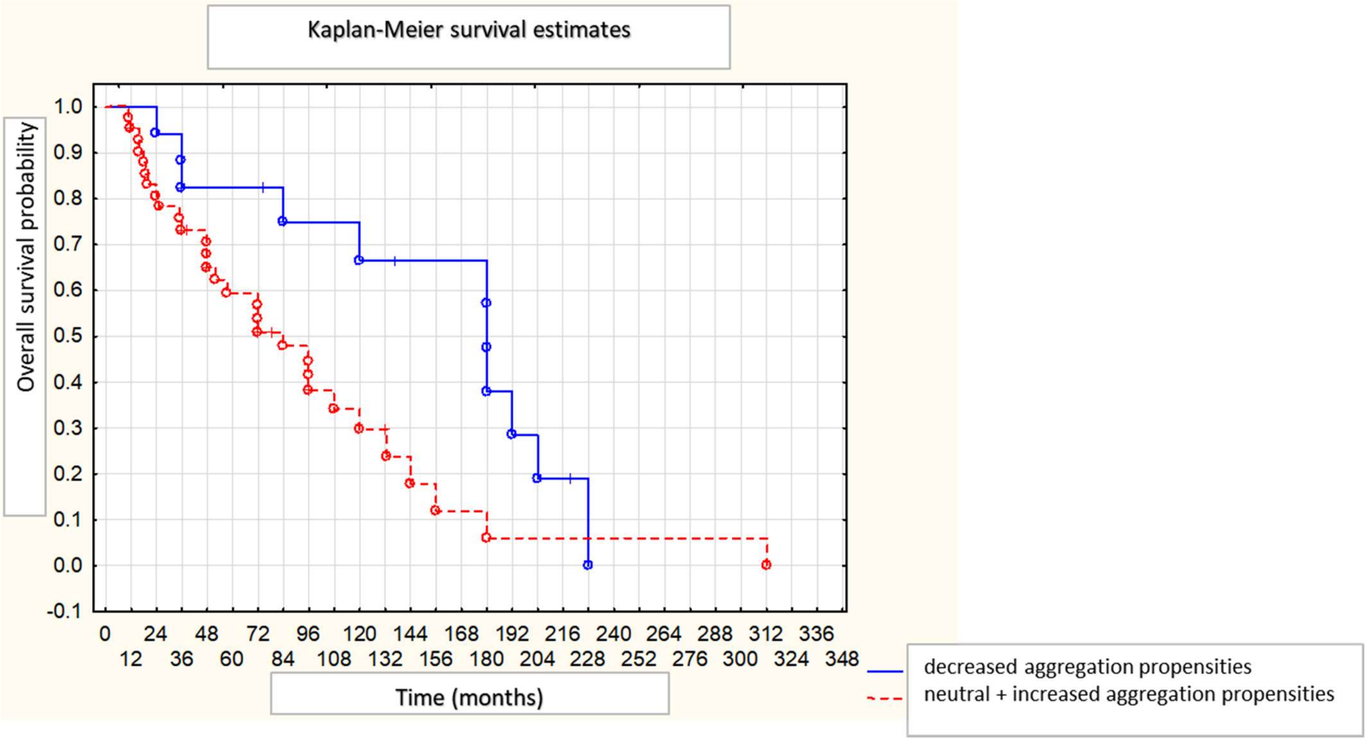

Figure S19. Comparison of overall survival between patients with decreased and neutral or increased TANGO aggregation propensities.
Log-rank test -2.51812 p = .01180

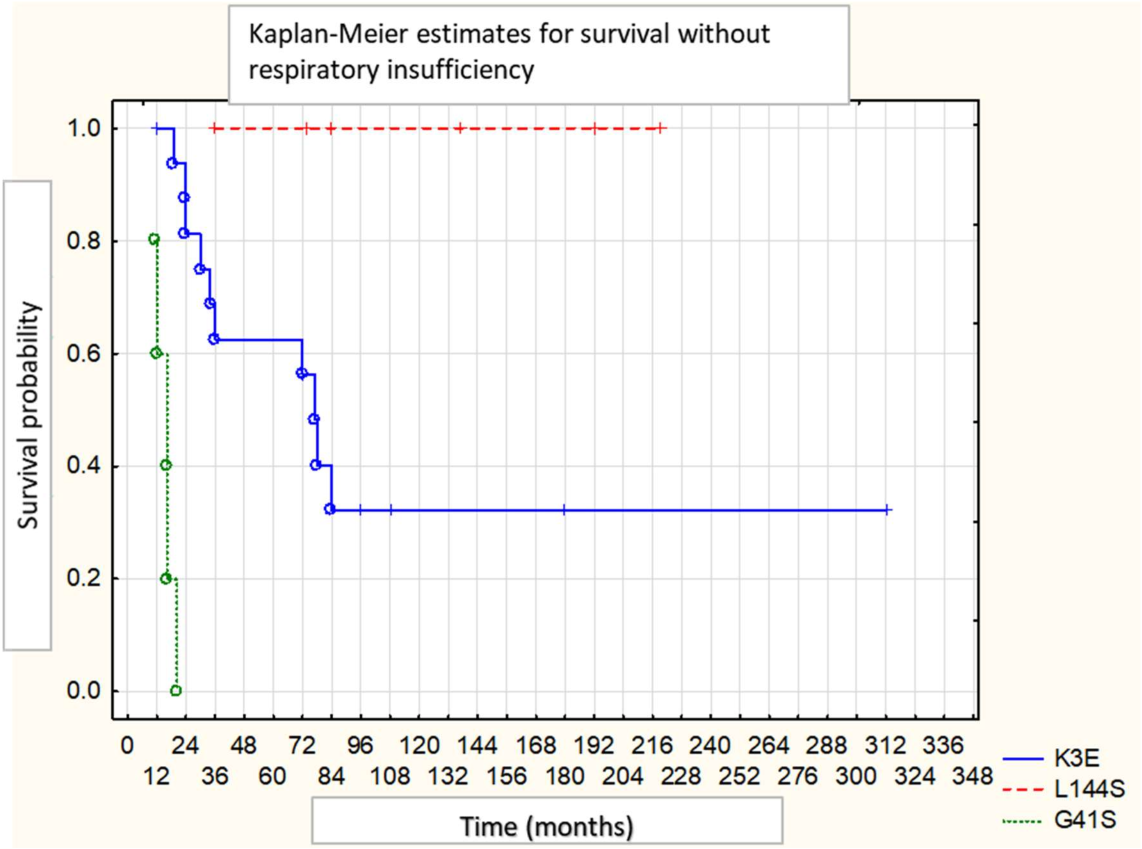

Figure S20. Comparison of survival without respiratory insufficiency between carriers of specific SOD1 mutations.

Chi2 = 16.8799 df = 2 p = .00022

K3E vs L144S Log-rank test: 2.483757 p = .01300

K3E vs G41S Log-rank test: -2.63528 p = .00841

L144S vs G41S Log-rank test: -3.10888 p = .00188

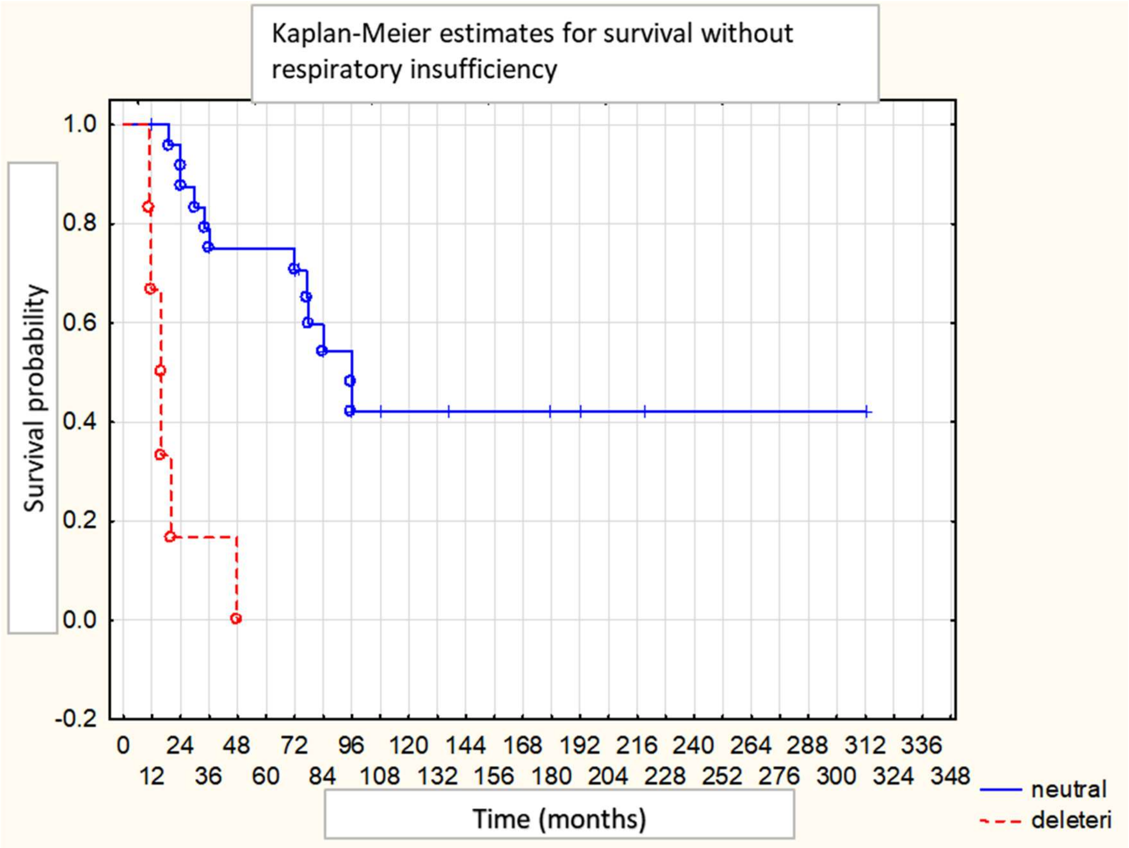

Figure S21. Comparison of survival without respiratory insufficiency between patients with neutral and deleterious PredictSNP results.

Log-rank test -3.00447 p = .00266

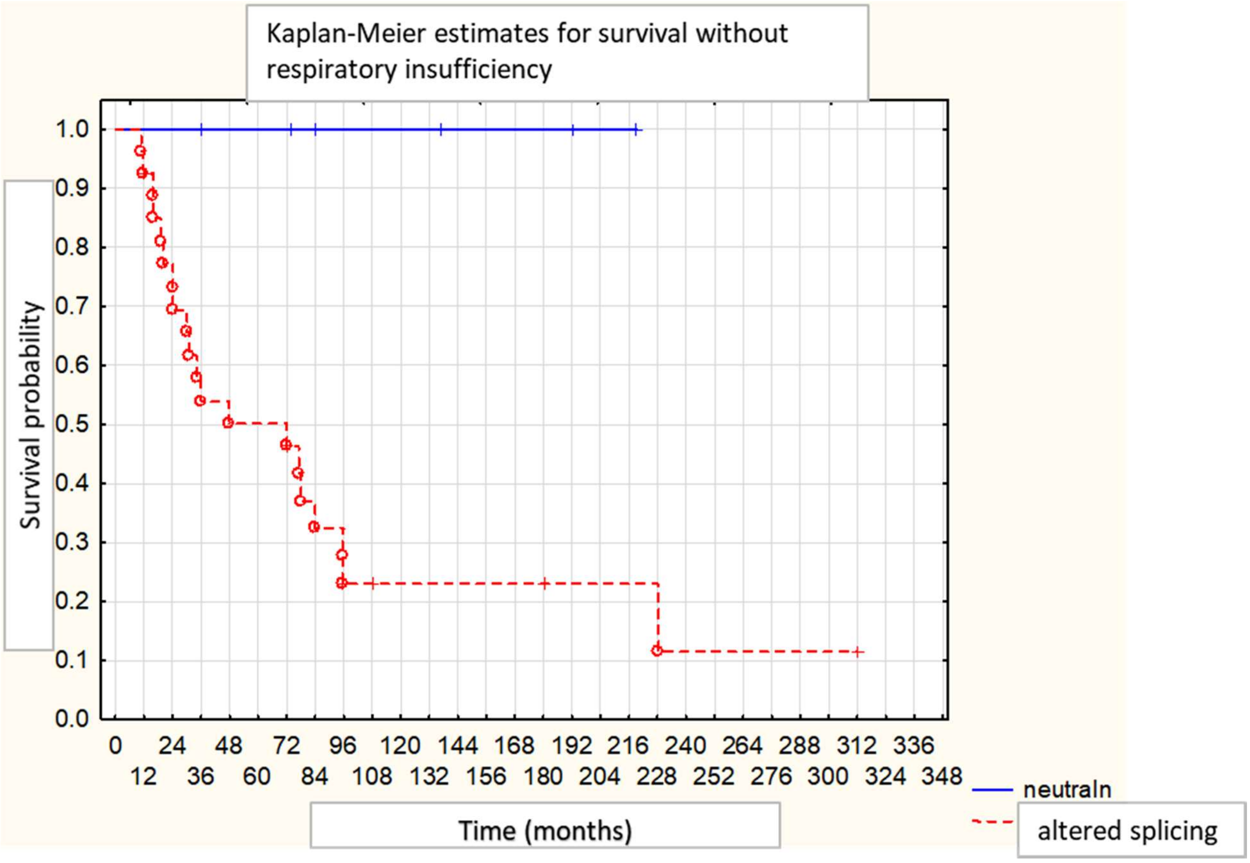

Figure S22. Comparison of survival without respiratory insufficiency between patients with neutral and altered splicing HSF 3.0 results.
Log-rank test -2.87837 p = .00400

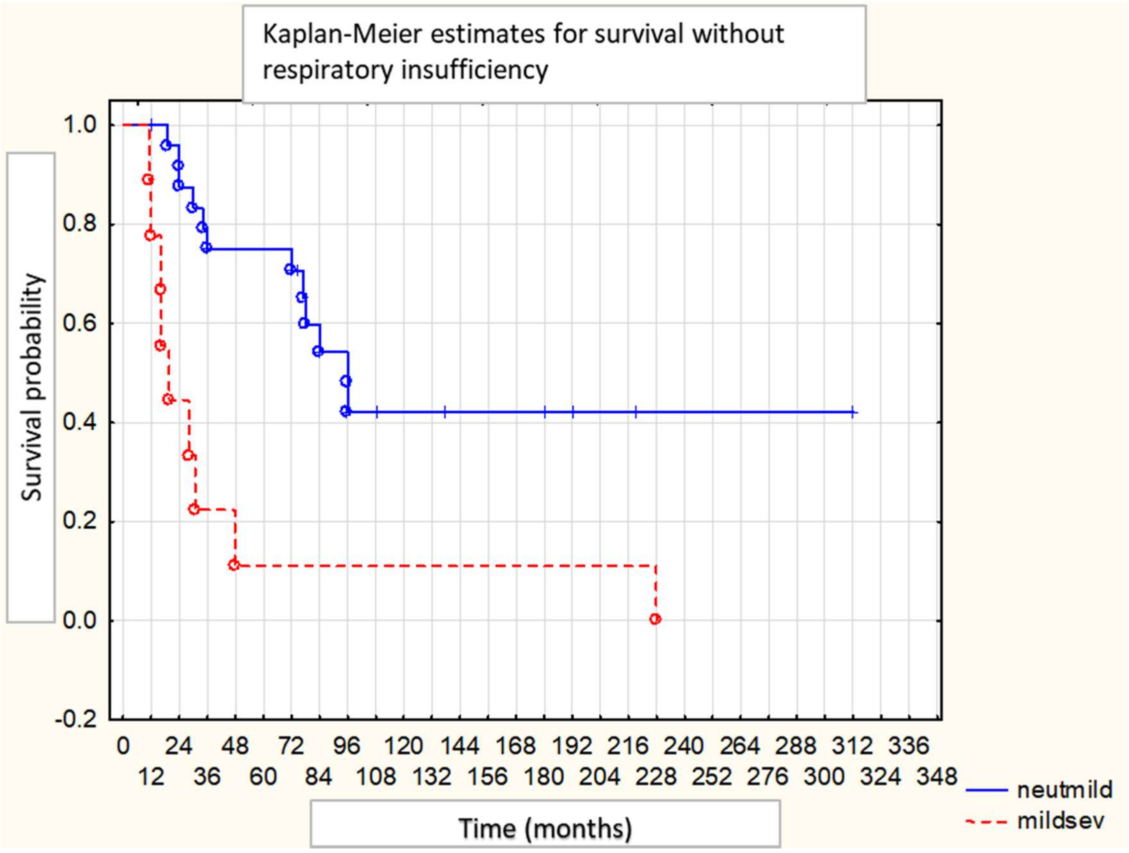

Figure S23. Comparison of survival without respiratory insufficiency between patients with neutral to mild and more severe than mild molecular modeling results.
Log-rank test -2.90978 p = .00362

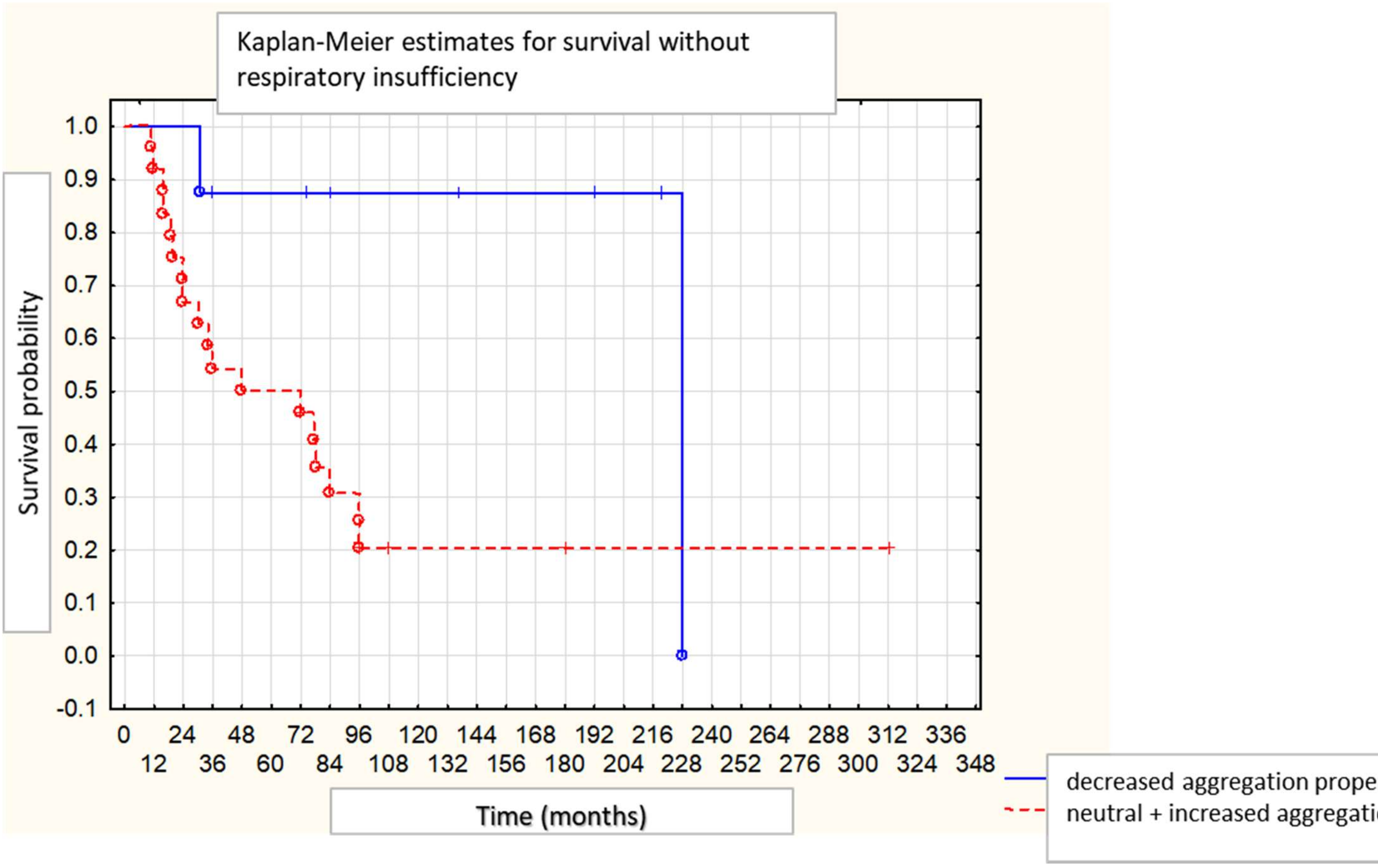

Figure S24. Comparison of survival without respiratory insufficiency between patients with decreased and neutral or increased TANGO aggregation propensities.
Log-rank test -2.51452 p = .01192

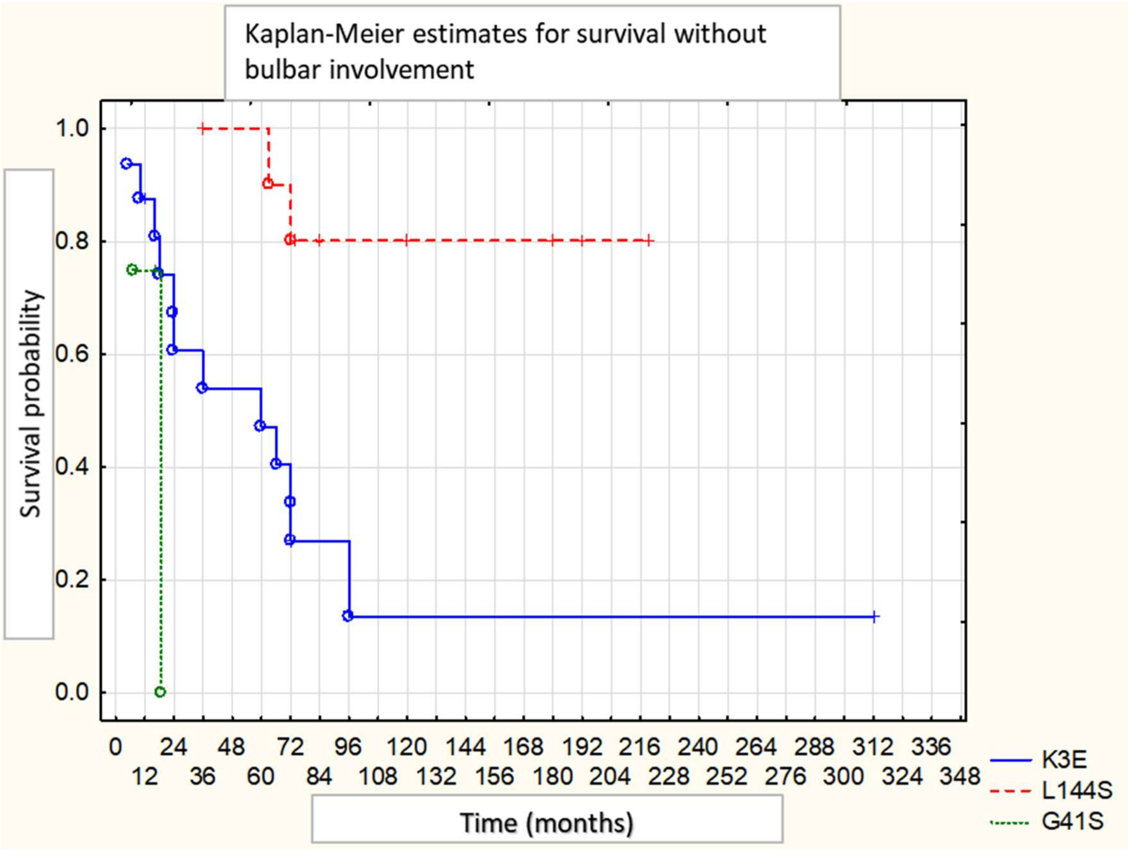

Figure S25. Comparison of survival without bulbar involvement between carriers of specific SOD1 mutations.
Chi2 = 10.6632 df = 2 p = .00484

K3E	vs	L144S	Log-rank test: 3.184531 p = .00145
K3E	vs	G41S	Log-rank test: -.765519 p = .44396
L144S	vs	G41S	Log-rank test: -1.95833 p = .05019

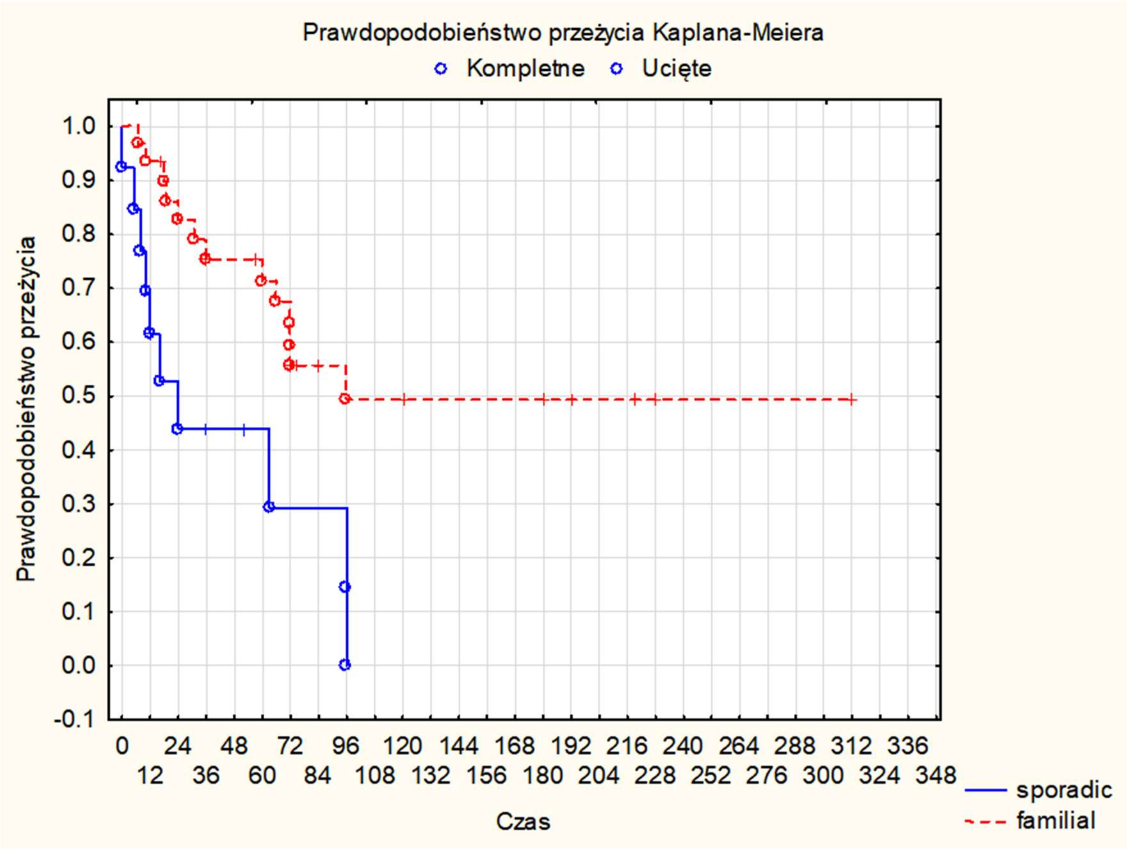

Figure S26. Comparison of survival without bulbar involvement between carriers of sporadic and familial SOD1 mutations.
Log-rank test 2.593049 p = .00951

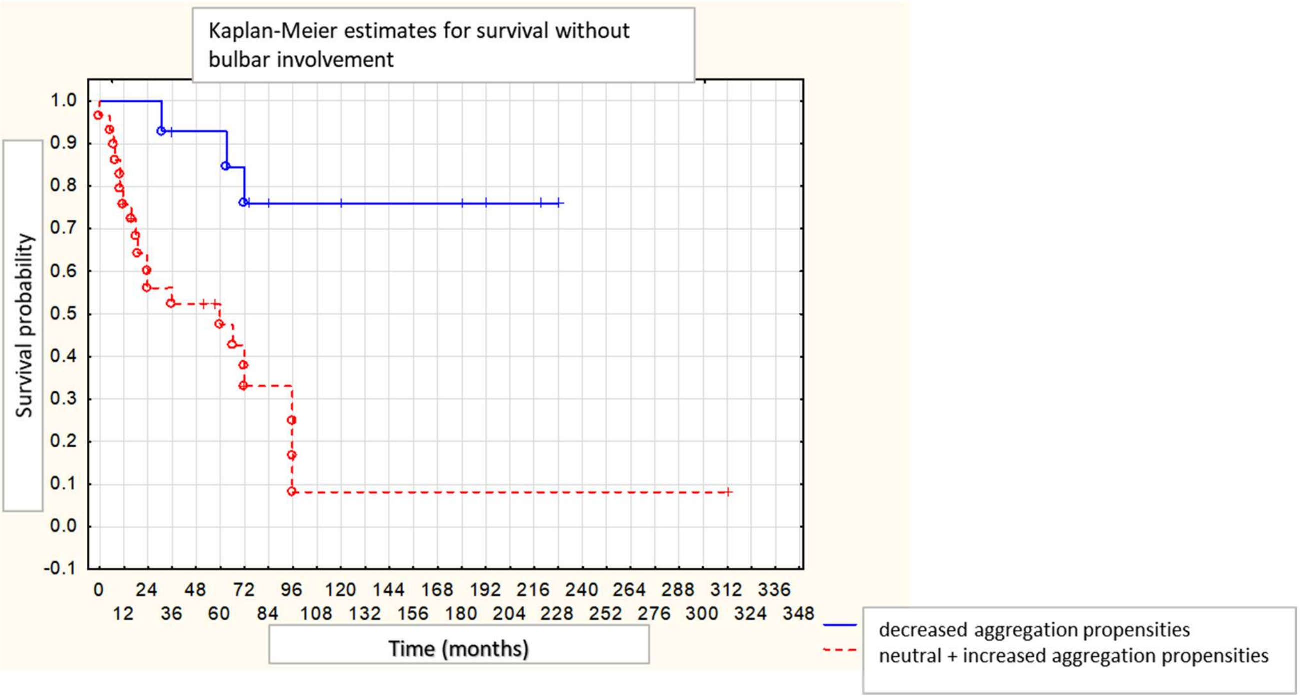

Figure S27. Comparison of survival without bulbar involvement between patients with decreased and neutral or increased TANGO aggregation propensities.
Log-rank test -3.52317 p = .00043

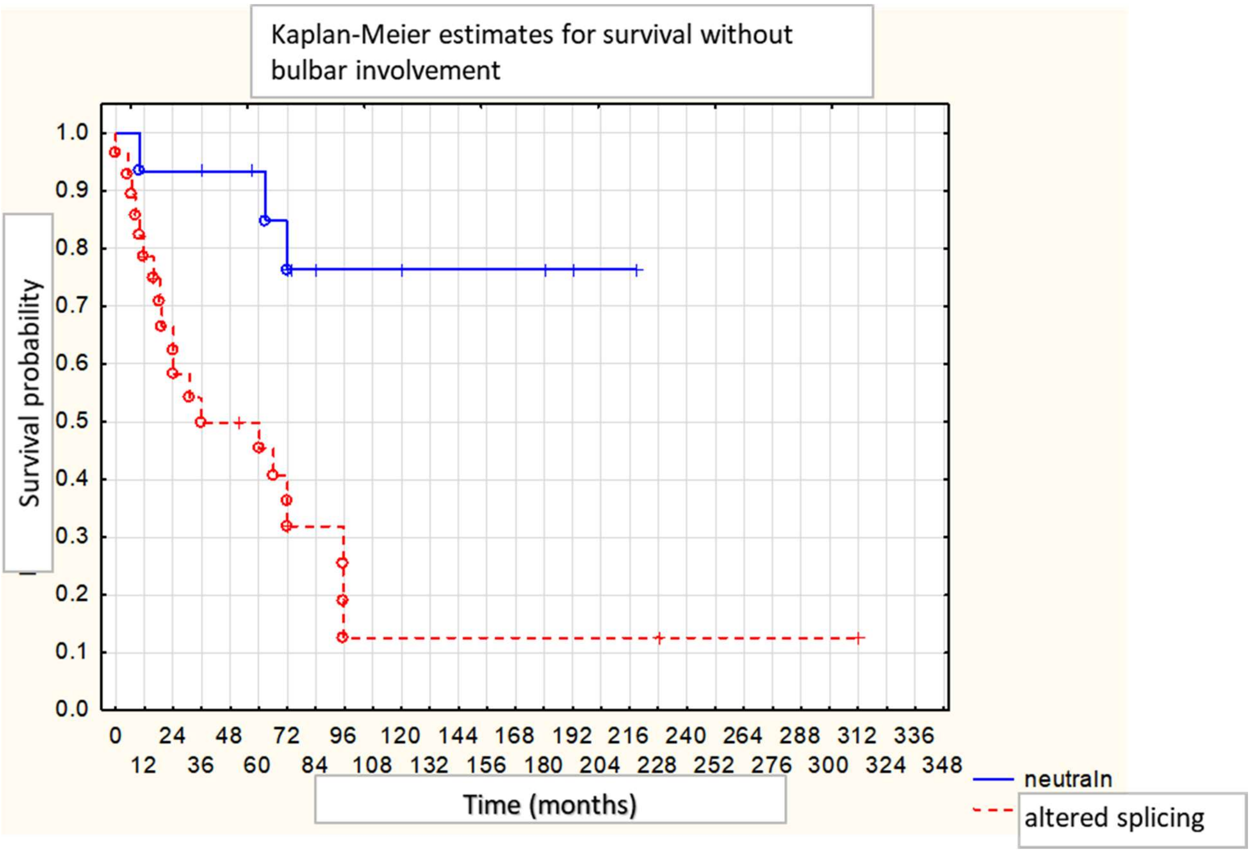

Figure S28. Comparison of survival without bulbar involvement between patients with neutral and altered splicing HSF 3.0 results.

Log-rank test -3.42419 p = .00062

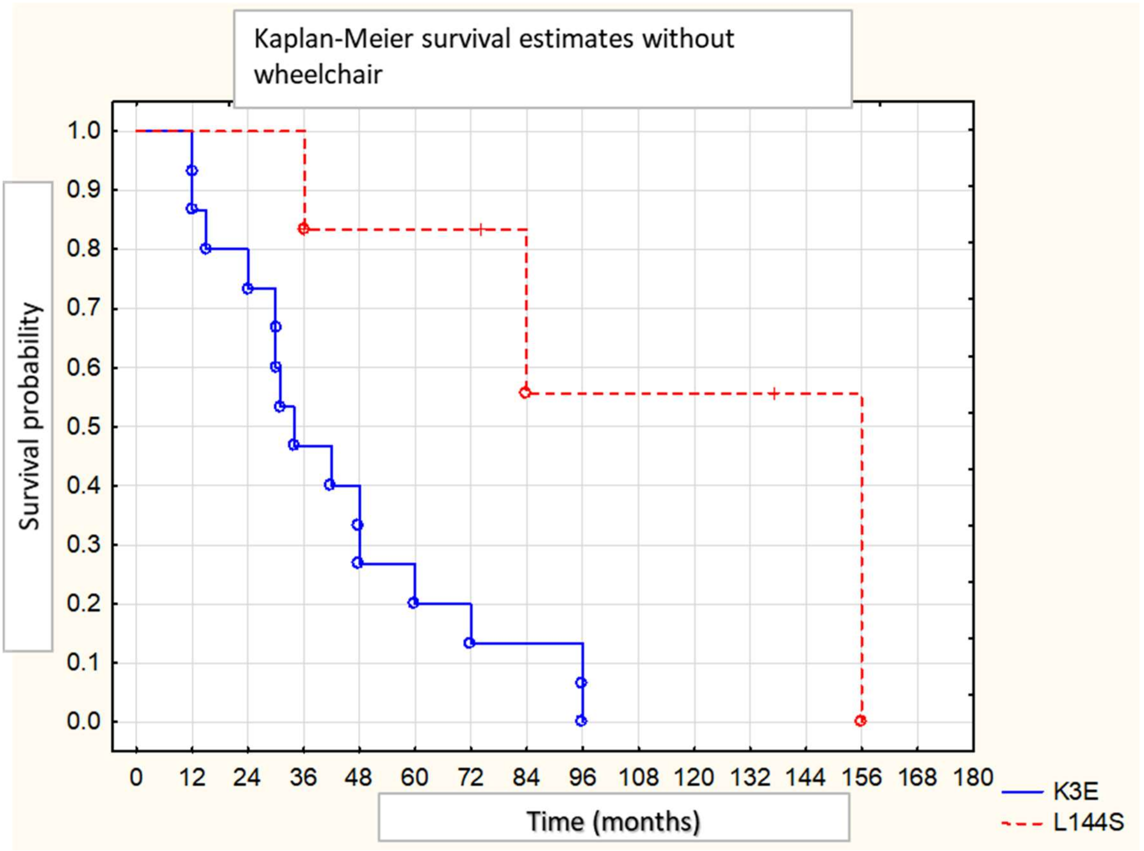

Figure S29. Comparison of survival without wheelchair between carriers of specific SOD1 mutations.

Log-rank test -2.95539 p = .00312

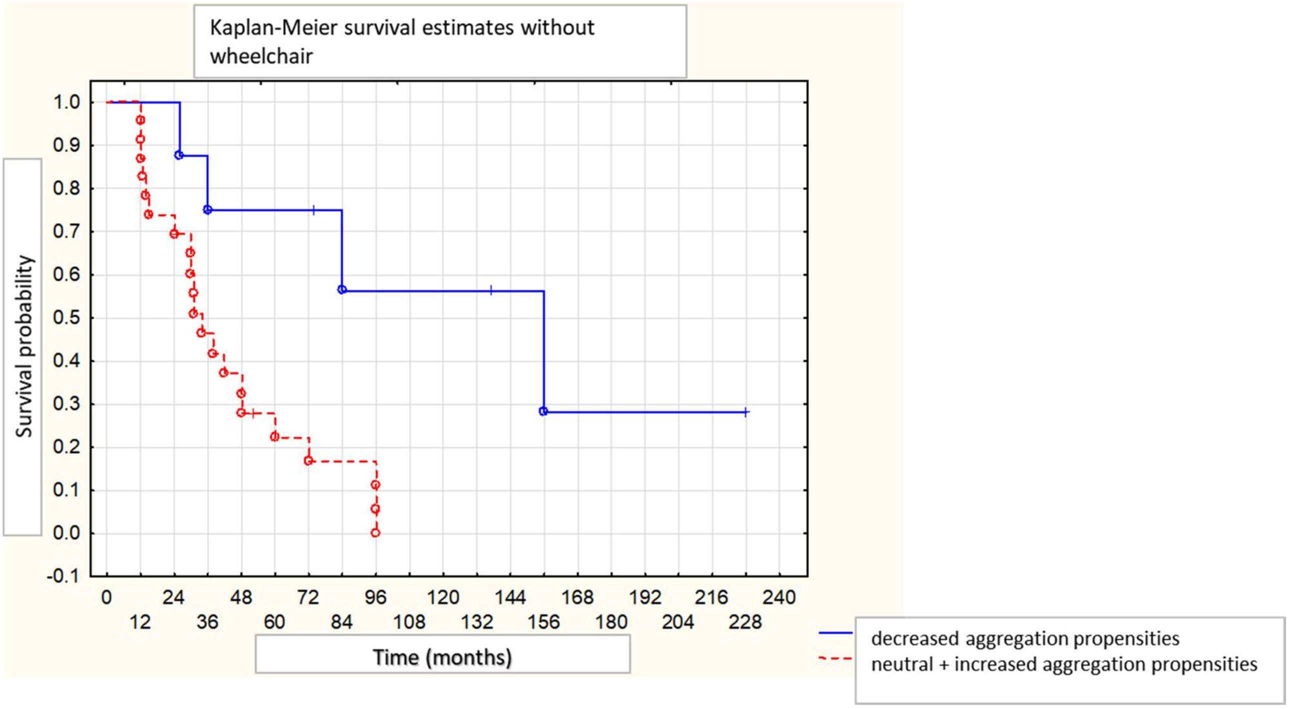

Figure S30. Comparison of survival without wheelchair between patients with decreased and neutral or increased TANGO aggregation propensities.

Log-rank test -2.95539 p = .00312

Table S1. Identified *SOD1* variants. Annotation and position coordinate from Variant Effect Predictor (ensembl.org) [9]

Traditional mutation name	HGVSc (NM_000454.4)	HGVSp (NP_000445.1)	POSITION (GRCh38)	REF	ALT	EXON	INTRON	cDNA position	Codons	Existing variation
	c.-64C>T		21:31659706	C	T	1		85		
	c.-59G>T		21:31659711	G	T	1		90		
	c.-7G>T		21:31659763	G	T	1		142		
**K3E**	c.10A>G	p.K4E	21:31659779	A	G	1		158	Aag/Gag	
**A4V**	c.14C>T	p.A5V	21:31659783	C	T	1		162	gCc/gTc	rs121912442
	c.72+19G>A		21:31659860	G	A		1			rs201574089
**W32***	c.98G>A	p.W33*	21:31663815	G	A	2		246	tGg/tAg	
**G37R**	c.112G>A	p.G38R	21:31663829	G	A	2		260	Gga/Aga	rs121912431
**G41S**	c.124G>A	p.G42S	21:31663841	G	A	2		272	Ggc/Agc	rs121912433
	c.169+50_169+56delTAAACAG		21:31663938-31663944	A	<DEL>		2			
**G72S**	c.217G>A	p.G73S	21:31666496	G	A	3		365	Ggt/Agt	rs121912455
	c.239+24A>T		21:31666542	A	T		3			
	c.239+34A>C		21:31666552	A	C		3			rs2234694
**N86S**	c.260A>G	p.N87S	21:31667278	A	G	5		408	aAt/aGt	rs11556620
**D90A**	c.272A>C	p.D91A	21:31667290	A	C	5		420	gAc/gCc	rs80265967
**G93C**	c.280G>T	p.G94C	21:31667298	G	T	5		428	Ggt/Tgt	rs121912437
**S105L**	c.317C>T	p.S106L	21:31667335	C	T	5		465	tCa/tTa	
**D109Y**	c.328G>T	p.D110V	21:31667346	G	T	5		476	Gac/Tac	rs567432143
**C111Y**	c.335G>A	p.C112Y	21:31667353	G	A	5		483	tGc/tAc	
**L126***	c.380T>A	p.L127*	21:31668493	T	A	5		528	tTg/tAg	rs121912454
**N139D**	c.418A>G	p.N140D	21:31668531	A	G	5		566	Aac/Gac	
**L144S**	c.434T>C	p.L145S	21:31668547	T	C	5		582	tTg/tCg	rs121912446
	c.*248A>C		21:31668826	T	C	5		861		

HGVSc - the HGVS coding sequence name; HGVSp - the HGVS protein sequence name

Table S2. ConSurf results of mutated aminoacids in SOD1 (ConSurf-DB Analysis for: 2C9V chain A)

POSITION	SEQ	SCORE	CONFIDENCE INTERVAL	MSA DATA	RESIDUE VARIETY
3	K	0.213	-0.210, 0.496	50/300	A,C,Q,S,K,R,Y,P,N,I,V,D
4	A	-1.284	-1.406,-1.223	57/300	A,T,I,V,G
32	W	1.054	0.731, 1.042	250/300	A,T,E,Q,H,R,K,S,M,Y,F,N,L,W,I,D,G,V
37	G	-0.348	-0.553,-0.210	273/300	G,T,Q,S,K,H,R,A,E,V,D,N
41	G	-0.755	-0.928,-0.691	287/300	R,N,S,G,D,A,T,E,C,Q,H,K
72	G	-0.721	-0.872,-0.624	290/300	D,G,P,L,E,A,T,H,R,K,S,Q
86	N	-1.266	-1.359,-1.223	300/300	I,V,P,L,N,E,T,A,K,R,H,M,S
90	D	0.729	0.309, 0.731	295/300	V,G,D,I,L,N,F,P,Y,Q,H,S,M,R,K,T,A,E
93	G	-0.901	-1.032,-0.815	300/300	G,D,N,A,T,E,Q,S,H,R,K

**105**	**S**	-0.078	-0.306, 0.016	298/300	D,V,G,N,P,L,T,Q,E,A,M,S,K,H,R,C
**109**	**D**	0.861	0.496, 1.042	230/300	L,N,P,S,D,V,Q,M,K,E,G,F,I,H,R,T,A,Y
**111**	**C**	0.861	-0.755,-0.553	290/300	K,M,Q,C,G,E,P,N,L,D,S,T,A,H,F,R,V
**126**	**L**	-0.300	-0.476,-0.210	286/300	Y,E,C,Q,S,M,K,H,G,D,F,P,N,L
**139**	**N**	-0.988	-1.081,-0.928	276/300	P,N,D,V,X,G,A,T,E,Q,S,M,K,H
**144**	**L**	0.053	-0.210, 0.153	276/300	F,P,L,W,I,V,T,A,E,Q,S,M,Y

1	2	3	4	5	6	7	8	9
	Variable			Average		Conserved		

SCORE: The normalized conservation scores.
CONFIDENCE INTERVAL: When using the bayesian method for calculating rates, a confidence interval is assigned to each of the inferred evolutionary conservation scores.
MSA DATA: The number of aligned sequences having an amino acid (non-gapped) from the overall number of sequences at each position.

Table S3. PredictSNP and NetDiseaseSNP results of mutated amino acids in SOD1

	**PredictSNP**	**MAPP**	**PhD-SNP**	**PolyPhen-1**	**PolyPhen-2**	**SIFT**	**SNAP**	**nsSNPAnalyzer**	**PANTHER**	**NetDiseaseSNP***
K3E	63%	64%	88%	67%	73%	53%	50%	65%	56%	0.53
A4V	87%	66%	86%	59%	81%	79%	62%	65%	74%	0.52
G37R	87%	82%	88%	74%	60%	79%	81%	63%	87%	0.90
G41S	55%	71%	86%	67%	56%	53%	62%	63%	66%	0.60
G72S	76%	48%	88%	59%	59%	79%	50%	63%	61%	0.28
N86S	87%	62%	82%	74%	68%	79%	62%	63%	68%	0.29
D90A	63%	48%	82%	67%	87%	68%	50%	65%	56%	0.73
G93C	87%	62%	88%	74%	65%	46%	56%	63%	84%	0.88
S105L	55%	65%	61%	59%	68%	53%	62%	65%	72%	0.74
D109Y	**60%**	70%	59 %	67%	87 %	79 %	56 %	-	61 %	0.81
C111Y	51%	57%	77%	67%	87%	79%	50%	0%	48%	0.96
N139D	51%	59%	73%	67%	63%	79%	55%	65%	73%	0.17
L144S	63%	68%	85%	67%	63%	45%	61%	63%	69%	0.75

**PredictSNP**	XX% expected accuracy	neutral	deleterious	https://loschmidt.chemi.muni.cz/predictsnp/
**NetDiseaseSNP**	Score	Neutral	Disease	http://www.cbs.dtu.dk/services/NetDiseaseSNP/

Table S4. Alternative splicing analysis by Human Splice Finder, Ex-Skip, BDGP splite Site Prediction

Traditional nomenclature	Human Splice Finder interperatation	EX-SKIP (wild type allele vs mutant allele)	BDGP splice site
**K3E**	Potential alteration of splicing	Both alleles have a comparable chance of exon skipping	no changes
**A4V**	Potential alteration of splicing	Allele wt has a higher chance of exon skipping than allele c.14C>T	no changes
	Probably no impact on splicing	Both alleles have a comparable chance of exon skipping	no changes
**W32***	Potential alteration of splicing	Allele c.98G>A has a higher chance of exon skipping than allele wt	loss of donor site score 0.67 (donor score cutoff 0.40)
**G37R**	Potential alteration of splicing	Allele c.112G>A has a higher chance of exon skipping than allele wt.	no changes
**G41S**	Potential alteration of splicing	Allele wt has a higher chance of exon skipping than allele c.124G>A.	no changes
	Probably no impact on splicing	Both alleles have a comparable chance of exon skipping.	no changes
**G72S**	Potential alteration of splicing	Allele wt has a higher chance of exon skipping than allele c.217G>A.	no changes
	This mutation has probably no impact on splicing	Both alleles have a comparable chance of exon skipping.	no changes
**N86S**	Potential alteration of splicing	Allele wt has a higher chance of exon skipping than allele c.260A>G.	no changes
**D90A**	Potential alteration of splicing	Allele 272A>C has a higher chance of exon skipping than allele wt.	no changes
**G93C**	Potential alteration of splicing	Allele wt has a higher chance of exon skipping than allele 280G>T.	no changes
**S105L**	This mutation has probably no impact on splicing	Allele wt has a higher chance of exon skipping than allele 317C>T.	new acceptor site score 0,47 (acceptor cutoff 0.40)
**D109Y**	Potential alteration of splicing	Allele c.328G>T has a higher chance of exon skipping than allele wt.	no changes
**C111Y**	This mutation has probably no impact on splicing	Allele wt has a higher chance of exon skipping than allele 335G>A.	no changes
**L126***	Potential alteration of splicing	Allele wt has a higher chance of exon skipping than allele 380T>A.	no changes
**N139D**	This mutation has probably no impact on splicing	Allele 418A>G has a higher chance of exon skipping than allele wt.	no changes
**L144S**	This mutation has probably no impact on splicing	Allele wt has a higher chance of exon skipping than allele 434T>C.	nothing change

Table S5. Aggrescan analysis (ttp://bioinf.uab.cat/aggrescan) of mutated amino acids in SOD1

Sequence Name:	a3v Sequence Average (a3vSA):	Number of Hot Spots (nHS):	Normalized nHS for 100 residues (NnHS):	Area of the profile Above Threshold (AAT):	Total Hot Spot Area (THSA):	Total Area (TA):	AAT per residue (AATr):	THSA per residue (THSAr):	Normalized a4v Sequence Sum for 100 residues (Na4vSS):
WT	-0.180	6	3.896	17.335	15.716	-24.854	0.113	0.102	-18.0
K3E	-0.183	6	3.896	17.060	15.441	-25.301	0.111	0.100	-18.3
A4V	-0.169	6	3.896	19.243	17.625	-23.108	0.125	0.114	-16.8
W32*	-0.016	2	6.250	6.378	5.705	-1.946	0.199	0.178	-8.3
G37R	-0.184	6	3.896	17.031	15.413	-25.559	0.111	0.100	-18.5
G41S	-0.178	6	3.896	17.371	15.718	-24.613	0.113	0.102	-17.9
G72S	-0.178	6	3.896	17.335	15.716	-24.613	0.113	0.102	-17.9
N86S	-0.173	6	3.896	17.552	15.716	-23.846	0.114	0.102	-17.4
D90A	-0.168	6	3.896	17.485	15.716	-23.054	0.114	0.102	-16.9
G93C	-0.172	6	3.896	17.498	15.716	-23.715	0.114	0.102	-17.3
S105L	-0.169	6	3.896	18.677	17.058	-23.180	0.121	0.111	-16.9
D109Y	-0.160	5	3.247	19.934	18.513	-21.859	0.129	0.120	-16.10
C111Y	-0.176	6	3.896	17.774	16.354	-24.299	0.115	0.106	-17.7
L126*	-0.173	5	3.968	13.043	11.425	-18.918	0.104	0.091	-17.5
N139D	-0.183	6	3.896	17.335	15.716	-25.388	0.113	0.102	-18.4
L144S	-0.191	6	3.896	16.626	15.008	-26.528	0.108	0.097	-19.1
